# A comparison of the presentations of males and females with autism spectrum disorder and those narrowly below the diagnostic threshold

**DOI:** 10.1177/13623613231190682

**Published:** 2023-08-22

**Authors:** Joanna M Tsirgiotis, Robyn L Young, Nathan Weber

**Affiliations:** Flinders University, Australia

**Keywords:** autism, female presentation, gender, sex

## Abstract

Little is currently known about the autism spectrum disorder (ASD)-related difficulties of females narrowly below the ASD diagnostic threshold and whether the disorder conceptualisation should be broadened to capture their presentations. We extracted fine-grained information from the assessment reports of 222 children who were either diagnosed with ASD (*n* = 156) or not diagnosed despite many ASD traits (*n* = 78). Females were less likely than males to meet Criterion B3 (restricted interests), and this was especially the case for subclinical (non-ASD) females. Non-ASD females lacked sufficient atypicality for diagnosis in several key domains, particularly Criterion A1 (social-emotional reciprocity). Evidence of sex/gender^1^-specific restricted interests and stereotypical behaviours was found. Many behaviours differed in the extent to which they predicted the ASD diagnostic result for males and females, perhaps suggesting that sex/gender influences how ASD-related behaviours are perceived. We discuss these findings in the context of underdiagnosis of ASD in females and propose adjustments to assessment protocols.

**Lay Abstract:**

Most research about autism spectrum disorder (ASD) in females has looked at autistic features in people who have already received diagnoses. Because our understanding of ASD has been shaped by the difficulties of males, females may experience different difficulties and may not meet the criteria for diagnosis because of a skewed concept of ASD. We extracted detailed information from the assessment reports of 222 children who were either diagnosed with ASD (156 children) or not diagnosed despite many ASD traits (78 children). Females were less likely to have restricted interests, especially females who did not receive an ASD diagnosis. Females who did not receive an ASD diagnosis tended to show more ability in social and emotional reciprocity than what would qualify them for a diagnosis. We also found sex-/gender-specific profiles of body use and speech mannerisms. Many behaviours were more closely linked with an ASD diagnosis for males and others for females, suggesting that behaviours may be interpreted differently depending on the child’s sex/gender. We discuss implications for assessing females for ASD in the context of this evidence.

Autism spectrum disorder (ASD) is diagnosed clinically, based on the presence of social communication difficulties and repetitive and restricted behaviours and interests (RRBIs), as well as associated behaviours and cognitive patterns defined in either the *Diagnostic and Statistical Manual for Mental Disorders* (DSM) or International Classification of Diseases (ICM). Assessment is typically conducted by collecting and evaluating data from various sources: referring parties, parents and caregivers, teachers or allied health professionals, observations made by the diagnostician(s) and the perspectives of client themselves. Assessment is very often augmented using psychometric tools to quantify the pervasiveness, variety and/or functional impact of ASD-related behaviours.

Growing evidence suggests that aspects of assessment for ASD may be invalid or insensitive for females, leading to substantial underdiagnosis (Loomes et al., 2017; O’Nions et al., 2023). Several hypotheses have been put forward to explain why autistic females may remain undiagnosed. For example, females may be less likely to be referred for specialist assessment (Øien et al., 2018) or to be considered as having ASD even if the severity of their symptoms is the same as that of males (Giarelli et al., 2010; Lai et al., 2015; Russell et al., 2011). Compounding this, females may present with difficulties that differ quantitatively and qualitatively from those of males, particularly in RRBIs (Lockwood Estrin et al., 2021).

To date, the vast majority of literature investigating the possibility of a distinctive female ASD presentation has followed the path of recruiting individuals with ASD diagnoses as participants. While this may be considered advantageous in other research, it presents a problem of circularity in this line of enquiry. Specifically, if indeed the ASD assessment instruments and conceptualisation are biased towards the typical male presentation (Kreiser & White, 2014; Rutter et al., 2003), only females whose presentation is sufficiently congruent with the male conceptualisation will receive ASD diagnoses. It remains unknown whether the remaining females fail to meet criteria because they are indeed not autistic (and thus should not be included in studies of the female ASD phenotype), or whether the criteria and diagnostic instruments are insensitive to the way their ASD is expressed (Lai et al., 2015).

A small number of studies has considered the presentations of individuals with subclinical ASD traits (i.e. individuals referred for ASD assessment and/or who achieve high scores on screening tools but are deemed ineligible for ASD diagnoses; Dworzynski et al., 2012; Wilson et al., 2016). These studies suggest that there may be asymmetry in how symptom severity and other factors influence the likelihood of ASD diagnosis for males and females. For example, greater behavioural and emotional difficulties have been found to increase the likelihood of ASD diagnosis more for girls than for boys, suggesting that girls require more atypicality in these areas to be considered autistic (Duvekot et al., 2017; Dworzynski et al., 2012). Parent-reported RRBIs have also been found to more strongly predict ASD diagnosis for boys than girls (Duvekot et al., 2017).

It remains unknown as to why autistic females may fail to be diagnosed correctly. Possibilities include (a) their difficulties are not considered clinically significant, (b) they do not demonstrate a broad-enough variety of ASD behaviours and/or (c) they do not present with difficulties that fit within the ASD diagnostic domains. Thus, it is important to examine behaviours at a fine-grained level where sex/gender differences are more likely to exist in order to fully appreciate the manifestation of ASD difficulties, rather than considering characteristics at the level of broad domains (i.e. social communicatoin and RRBIs; Hiller et al., 2014; Lai et al., 2015).

The level of atypicality reported both in general and in specific areas may vary significantly according to the source of information (Hiller et al., 2014; Mayes & Lockridge, 2018; Tsirgiotis et al., 2022a). It is now understood that ASD may present inconsistently across different environments, and therefore, discrepancies in what is reported may be expected. These discrepancies may be larger for females because of a proclivity to engage in camouflaging or masking ASD-related difficulties in social environments (Hull et al., 2020). As a result, atypicality reported by teachers may be less significant for autistic girls than for boys (Hiller et al., 2014). Social camouflaging may also influence the ASD behaviours observed by a diagnostician at the time of assessment and result in discrepancy between diagnostic observations and parent report (Mayes & Lockridge, 2018). Although diagnosticians surveyed by Tsirgiotis et al. (2022a) reported that it is challenging to reconcile contrasting information from different sources, the relative influence of each report on diagnostic outcomes for males and females remains unexplored.

In light of the aforementioned gaps in our knowledge, the present study examined the ASD presentations of children narrowly below the ASD diagnostic threshold; specifically, those who were suspected of having ASD and were found to have many ASD traits but who did not fully meet criteria at assessment. Fine-grained data regarding behaviours were extracted from the parent-report and teacher-report data and diagnostic observations documented in the children’s ASD assessment reports of 222 children (*n* = 98 females). The following research questions were posed:

1. Which criteria did females commonly fail to meet, and what was the nature of their difficulties in these areas (if any)?
2. What were the differences in the ASD-related features of females with ASD and females under the threshold (non-ASD)? Were these the same for males?
3. Which behaviours predicted ASD diagnosis, and was this the same for males and females?

## Community involvement

The motivation behind this research focus was, in part, the result of consultation with parents of autistic children. The authors have personal experience as family members and friends of autistic people, in addition to clinical and research experience. While autistic children participated in this study, their parents were not directly involved in the research design.

## Method

### Participants

Data were extracted from the ASD diagnostic assessment reports of 222 children (*n* = 98 females and *n* = 124 males) who had undertaken an ASD assessment and either (a) received an ASD diagnosis, hereafter referred to as the ASD group, or (b) did not receive an ASD diagnosis, despite the presence of ASD traits the non-ASD group. Participants were excluded if their cognitive ability was in the range of intellectual disability (full scale IQ ⩽70).^2^ The ASD group comprised 156 children (females: 50.0%, *n* = 78; males: 50.0%, *n* = 78).

A total of 79 children were identified as having received a negative ASD assessment result. To restrict the sample to children with subclinical ASD traits, the following inclusion criteria were applied:

1. At assessment, no alternative diagnostic explanation was given to *entirely* explain the presenting difficulties (*n* = 10 excluded);
2. At assessment, the child was deemed to at least partially meet one social communication criterion and one RRBI criterion (*n* = 3 excluded); and
3. The child had not received a diagnosis of ASD since the time of their assessment and data collection.

After these exclusion criteria were applied, our non-ASD sample comprised 66 children (females: 30.3%, *n* = 20; males: 69.7%, *n* = 46).

#### Assessment process

Participants were assessed for ASD by psychologists and/or speech pathologists with specialist training in ASD assessment at a large private clinic specialising in neurodevelopmental disorders. Following assessment, children diagnosed with ASD were then referred and registered with the local autism organisation wherein independent ASD diagnosticians verified and endorsed the diagnosis from the reports provided. The assessment procedure employed at this clinic is compliant with best practice guidelines for ASD assessment in Australia (Whitehouse et al., 2018). This clinic is well known for upholding the core tenants of excellence in ASD assessment: reliance on current scientific evidence and employing an individual- and family-oriented approach. Assessments are conducted by either a single psychologist diagnostician or a psychologist and speech pathologist diagnosticians. Assessments are of approximately 3-hours duration and include a detailed parent/caregiver interview, diagnostic observations and examination of teacher observations and any referral information (including previous assessment reports and correspondence pertaining to the child’s development). DSM-5 criteria are used as the foundation for diagnoses. At this clinic, parent interviews are lengthy (at least 2 hours) and are based on the Autism Diagnostic Interview-Revised (Lord et al., 1994). As such, parent interviews collect a comprehensive developmental history and involve discussion of all features currently associated with ASD. Diagnostic observations are conducted for the entire period during which the diagnostician is with the child. Observations are reported in an unstructured manner within each criterion or as part of the documentation of findings using a structured assessment instrument (e.g. Autism Diagnostic Observation Schedule; Lord et al., 2012; or Autism Detection in Early Childhood; Young, 2007). The child’s current teacher’s feedback is collected via responses on a structured, open-ended questionnaire, directly reflective of DSM-5 criteria.^3^

In Australia, where this research took place, it is recommended that standardised psychometric tools are used to support diagnostic reasoning. A variety of tools are used at this clinic and are selected based on clinician preference and the age and cognitive ability of the child.^4^

The diagnosticians produce detailed assessment reports summarising diagnostic information from all sources and categorised by DSM-5 criterion. These reports are compliant with the requirements for registration with the local autism organisation and with the Australian National Disability Insurance Scheme.

### Research procedure

Informed consent for the use of assessment information in research was obtained at intake from parents and children if appropriate. Ethics approval was granted by the authors’ tertiary institution. We identified eligible children from records of assessments conducted between September 2013 and March 2019. These children were assessed by one or two of seven diagnosticians with between 3 and 20 years of experience. The first author performed the initial data extraction from the ASD assessment reports and referral material: background and demographic information and the extent to which each criterion was met – (0) *not met*, (1) *partially met*, (2), *met*. To address our specific research questions, fine-grained data regarding the presence of specific behaviours and difficulties were collected from three sources of reporting (parent interview, teacher report and diagnostician observation) and rated as either (0) *absent* or (1) *present*.^5^

Parent-report and diagnostician observation data were available for all children, but teacher-report data were only available for 71.6% of children (64.3% of females, 77.4% of males). Following data collection, an independent rater, blind to the study aims, re-coded 10% of the data, and there was between 78.3% and 100% agreement across variables (*M* = 86.8%). Intra-rater reliability checks conducted on 15% of the data showed consistency between 87% and 100% across variables. Variables with less than 85% agreement were excluded from the analysis.

### Data analysis

A Bayesian parameter estimation approach was used for data analysis. The advantages of such an approach are discussed elsewhere (Tsirgiotis et al., 2022a, 2022b). Importantly, unlike traditional statistical methods, Bayesian analyses produce a distribution of the most credible values (posterior distribution) for a parameter. These are summarised here using highest density intervals (HDIs), within which lie the most credible values of a parameter. We use an HDI criterion of 80%, that is, the probability of the HDI containing the true value of a parameter was set at 80%. We can have 80% confidence that the true value of a parameter is included in the HDI_80%_, and therefore, the breadth of the HDI indicates the degree of uncertainty in an estimate. The HDI is interpreted with the aid of a Bayesian region of practical equivalence (ROPE), which captures the parameters of no meaningful (negligible) difference, or practical equivalence, to the null. If the HDI_80%_ lies entirely within the ROPE, we can have 80% confidence of no meaningful effect (P_(negligible)_ ≈ 100%). Conversely, if the HDI_80%_ lies entirely outside the ROPE, we can have 80% confidence in a meaningful effect (P_(meaningful)_ ≈ 100%). Finally, if the HDI_80%_ only *partially* overlaps the ROPE, there is equivocal difference regarding accepting the null at the 80% confidence level. However, the character of the posterior distribution relative to the ROPE reflects the probability of a meaningful effect.

Bayesian logistic regression analysis was applied, and log odd ratios used as the basis for inference, with relative risk reported to aid interpretation. The assessment decision (i.e. positive or negative ASD result) and the child’s sex/gender were defined as predictor variables (between groups). The presence/absence of any given ASD behaviour was coded in binary form.^6^ Age (centred and scaled to *SD* = 1) was included as a covariate. The interaction between assessment result and sex/gender on whether any particular behaviour was reported was of particular interest in the present study. In referring to interactions, relative risks are reported. However, in some instances, the relative risk denominators differed considerably between males and females, rendering them difficult to compare. In these cases, it is more valuable to consider proportion differences reported in the tables (i.e. the proportion of ASD males for whom the behaviour was reported minus the proportion of non-ASD males for whom the behaviour was reported; and likewise for females).

Simple main effects were also examined in order to identify whether ASD or non-ASD children contributed more towards any given sex/gender difference (i.e. probability for ASD males minus females; probability for non-ASD males minus females). Noteworthy simple main effects are displayed in the tables and in the text. Fine-grained data were collected on 72 variables from parent report, 20 from diagnostic observation and 13 from teacher report. In the Results section, we present the variables in which meaningful effects were found. A complete list of the variables and results is presented in the Supplementary material.

## Results

### Age at assessment and cognitive ability

Children’s ages ranged from 1 year and 6 months to 17 years and 10 months (*M* = 8.23, *SD* = 3.89 years). An analysis of variance revealed meaningful differences in the ages, but not cognitive ability of children in each group (Table 1), with females tending to be older than males in each group. Standardised information regarding cognitive ability was available for 70.4% of females and 59.7% of males (60.9% of ASD children and 72.7% of non-ASD children). This information had been collected at the time of (or prior to) diagnostic assessment, using either the Wechsler Intelligence Scale for Children (Wechsler, 2003, 2014) or Wechsler Preschool and Primary Scale of Intelligence (Wechsler, 2002, 2012). Results of incomplete cognitive assessments were prorated (19.6% of cases).

**Table 1.** Age and intellectual ability of children by sex/gender and assessment result.

Group	Variable	Males	Females	All	Male vs females *d*, HDI_80%_, P_(meaningful)_
ASD children	*n*	78	78	156	
	Age (years)				
	*M* (*SD*)	6.90 (3.26)	9.51 (4.32)	8.19 (4.06)	–0.67, [−0.87, –0.47], 100%
	Range	2.08–16.58	1.83–17.83	1.83–17.83	
	IQ				
	*M* (*SD*)	96.88 (12.11)	96.67 (15.23)	96.94 (14.15)	0.01, –[0.22, 0.25], 43%
	Range	76–136	70–131	70–136	
Non-ASD children	*n*	46	20	66	
	Age (years)				
	*M* (*SD*)	8.15 (2.96)	8.53 (4.53)	8.31 (3.48)	–0.10, [−0.45, 0.23], 50.3%
	Range	2.00–14.16	1.50–17.83	1.50–17.83	
	IQ				
	*M* (*SD*)	95.38 (15.19)	97.00 (13.16)	95.73 (14.39)	–0.10, [−0.41, 0.19], 50%
	Range	70–137	78–126	70–137	
All	*n*	124	98	222	
	Age (years)				
	*M* (*SD*)	7.33 (3.21)	8.31 (3.48)	8.23 (3.89)	
	Range	2.00–16.58	1.50–17.83	1.50–17.83	
	IQ				
	*M* (*SD*)	96.07 (13.52)	95.73 (14.39)	96.47 (14.19)	
	Range	70–137	70–131	70–137	
ASD vs non-ASD *d*, HDI_80%_, P_(meaningful)_	Age (years)	0.32, [0.08, 0.53], 89.7%	0.25, [−0.04, 0.53], 75.4%		
	IQ	0.10, –[0.15, 0.37], 50.3%	−0.02, [−0.32, 0.26], 36.1%		

HDI: highest density interval; ASD: autism spectrum disorder; IQ: intelligence quotient.

Twenty-three percent of children presented with cooccurring language delay (ASD children: 41.0% of males and 7.7% of females; non-ASD children: 21.7% of males and 15.0% of females). Other psychiatric diagnoses (e.g. depression, attention-deficit/hyperactivity disorder) had been made for 19.8% of children prior to ASD assessment (ASD children: 20.5% of males and 25.6% of females; non-ASD children: 8.7% of males and 20.0% of females).

### Referral information

Information regarding family history of ASD was available for 85.0% of children. There was strong evidence that children who received ASD diagnoses had a higher probability of having a family history of ASD (log odds ratio [LOR] = 0.70, HDI_80%_ = [0.27, 1.13], P_(meaningful)_ = 96.4%). In general, females had a higher probability of having a family member with ASD (LOR = −0.85, HDI_80%_ = [−1.28, 0.41], P_(meaningful)_ = 98.8%), but a positive family history was more strongly associated with receiving an ASD diagnosis for males (LOR = 0.76, HDI_80%_ = [−0.13, 1.59], P_(meaningful)_ = 83.5%).

A child had a higher probability of being referred by a professional (doctor, allied health professional or the school) than by their family if they were ultimately diagnosed with ASD (LOR = 1.02, HDI_80%_ = [0.48, 1.57], P_(meaningful)_ = 98.4%) and if they were male (LOR = 0.57, HDI_80%_ = [0.03, 1.11], P_(meaningful)_ = 86.9%).

Females had a higher probability of having received a previous psychiatric diagnosis than males (LOR = −0.63, HDI_80%_ = [−1.20, 0.08], P_(meaningful)_ = 88.7%). Similarly, children subsequently diagnosed with ASD had a meaningfully higher probability of having received a previous diagnosis than children who were not diagnosed with ASD (LOR = 0.73, HDI_80%_ = [0.18, 1.31], P_(meaningful)_ = 93.0%).

#### Levels of ASD-related atypicality reported

Table 2 presents the proportion of ASD behaviours for which ASD-related atypicality was reported for each group of children by each report source.^7^

**Table 2.** Proportion of ASD behaviours for which ASD-consistent atypicality was reported.

Source	Domain	Proportion of behaviours with atypicality reported per group (%)			
		ASD		Non-ASD	
		Males	Females	Males	Females
		*n* = 78	*n* = 78	*n* = 46	*n* = 20
Parent report	SC	46.4	47.1	22.5	29.5
	RRBI	42.8	40.9	17.0	22.6
Diagnostician observation	SC	38.8	33.2	16.9	14.6
	RRBI	21.3	15.9	7.3	6.3
Teacher report	Data availability^a^	*n* = 55	*n* = 51	*n* = 41	*n* = 12
		70.5%	65.4%	89.1%	60.0%
	SC	63.0	40.8	36.6	30.6
	RRBI	32.2	16.8	16.4	13.1

SC = social communication; RRBI = restricted and repetitive behaviours and interests; ASD: autism spectrum disorder.

a Parent report and diagnostician observation data were available for all children.

Sex/gender differences in the probability that atypicality was raised by each source was investigated using Bayesian hierarchical conditional logistic regressions (Table 3). As expected, children who received ASD diagnoses were more likely to have atypicality reported across all domains and sources of reporting. ASD males had a higher probability of having atypicality reported by teachers than ASD females across both domains. Parent-reported atypicality was more strongly associated with ASD result for males than for females, and there was weaker evidence of this for teacher report.

**Table 3.** Results of logistic regressions for the level of ASD-consistent atypicality by sex/gender and assessment according to each source.

Source: domain		Effect of Ax. result		Effect of sex/gender		Ax. result × sex/gender interaction		ASD: M–F		Non-ASD: M–F	
		LOR [HDI_80%_]	P_(meaningful)_	LOR [HDI_80%_]	P_(meaningful)_	LOR [HDI_80%_]	P_(meaningful)_	LOR [HDI_80%_]	P_(meaningful)_	LOR [HDI_80%_]	P_(meaningful)_
Parent	SC	**1.00 [0.90, 1.10]**	**1.00**	**–0.24 [−0.34, –0.14]**	**–0.96**	**0.34 [0.14, 0.54]**	**0.94**	−0.07 [−0.17, 0.03]	−0.34	**–0.41 [−0.58, –0.23]**	**–0.99**
	RRBI	**1.15 [1.02, 1.27]**	**1.00**	−0.19 [−0.31, –0.06]	−0.81	**0.37 [0.11, 0.61]**	**0.91**	−0.00 [−0.12, 0.11]	−0.14	**–0.37 [−0.60, –0.16]**	**–0.94**
Diagnost. obs.	SC	**1.15 [1.00, 1.30]**	**1.00**	0.19 [0.04, 0.34]	0.77	−0.07 [−0.38, 0.23]	−0.45	0.15 [0.01, 0.28]	0.69	0.22 [−0.05, 0.49]	0.71
	RRBI	**1.39 [1.02, 1.74]**	**1.00**	0.40 [0.04, 0.75]	0.86	−0.37 [−1.07, 0.32]	−0.70	0.20 [−0.04, 0.46]	0.71	0.58 [−0.12, 1.19]	0.83
Teacher	SC	**0.82 [0.60, 1.03]**	**1.00**	**0.57 [0.36, 0.79]**	**1.00**	0.52 [0.09, 0.96]	0.89	**0.83 [0.61, 1.06]**	**1.00**	0.32 [−0.07, 0.67]	0.77
	RRBI	**0.75 [0.46, 1.02]**	**1.00**	**0.64 [0.36, 0.93]**	**0.99**	0.43 [−0.12, 0.99]	0.77	**0.85 [0.58, 1.11]**	**1.00**	0.42 [−0.05, 0.92]	0.81

SC: social communication; RRBI: repetitive and restricted behaviours and interests; LOR: log odds ratio; ASD: autism spectrum disorder; Ax.: Assessment; HDI: highest density interval; ROPE: region of practical equivalence.

Positive LORs (ASD assessment result) = greater probability of being reported if the assessment result was positive for ASD; positive LORs (sex/gender) = greater probability of being reported for males. Rows in boldface indicate that the HDI_80%_ fell entirely outside the ROPE. P_(meaningful)_ = probability that the true difference fell outside the ROPE and in the observed direction.

Across both social communication and RRBIs, parents were approximately equally likely to report atypicality for males and females who received ASD diagnoses. However, for non-ASD children, parents of females were more likely to report atypicality for their daughters in both domains than parents of males. There was weak evidence of a higher probability of diagnosticians observing atypical behaviour for males than for females in both the social communication and RRBI domains, regardless of ASD result. This was also the case for teacher-reported RRBIs. However, there was strong evidence that, among children diagnosed with ASD, teachers were more likely to report atypicality for males across both domains.

#### Criterion fulfilment

The probability that each criterion was met (compared to partially met or not met) was analysed using conditional logistic regression, with sex/gender and assessment decision (i.e. assessment result) specified as predictor variables (Table 4).

**Table 4.** Logistic regression effect of assessment result, sex/gender and their interaction on criteria met.

	Effect of Ax. result	Effect of sex/gender		Ax. result × sex/gender interaction	
Criterion	LOR [HDI_80%_]^a^	LOR [HDI_80%_]	P_(meaningful)_	LOR [HDI_80%_]	P_(meaningful)_
Domain A	9.48 [6.71, 12.01]	0.14 [−2.11, 2.29]	0.51	−0.14 [−4.37, 4.18]	−0.51
A1	7.67 [4.79, 10.62]	0.57 [−0.38, 1.70]	0.72	−0.58 [−2.90, 0.92]	−0.65
A2	8.32 [4.90, 12.39]	0.01 [−0.72, 0.84]	0.40	−0.01 [−1.47, 1.39]	−0.43
A3	7.83 [4.36, 11.67]	0.02 [−0.72, 0.84]	0.43	−0.04 [−1.56, 1.32]	−0.46
Domain B	8.68 [6.05, 11.24]	−0.07 [−2.09, 1.95]	−0.49	−0.03 [−3.99, 3.93]	−0.49
B1	1.63 [1.21, 2.08]	0.22 [−0.12, 0.62]	0.65	−0.01 [−0.65, 0.63]	−0.40
B2	2.49 [2.02, 2.95]	−0.21 [−0.64, 0.16]	−0.63	−0.05 [−0.77, 0.58]	−0.46
B3	2.01 [1.47, 2.51]	0.62 [0.06, 1.10]	0.91	−0.03 [−0.78, 0.72]	−0.44
B4	3.12 [2.59, 3.65]	−0.40 [−0.88, 0.10]	−0.80	−0.01 [−0.74, 0.71]	−0.41

ASD: autism spectrum disorder; Ax.: Assessment; LOR: log odds ratio; HDI: highest density interval.

Criterion A1: deficits in social-emotional reciprocity; A2: deficits in nonverbal communication behaviours; A3: deficits in developing, maintaining and understanding relationships; B1: stereotyped/repetitive motor movements, use of objects or speech; B2: insistence on sameness, routines or ritualised behaviour; B3: restricted and fixated interests; B4: hyper-/hypo-reactivity to sensory input (DSM-5). Positive LORs (ASD assessment result) = greater probability of being met if the result was positive for ASD; positive LORs (sex/gender) = greater probability of being met for males. P_(meaningful)_ = probability that the true difference fell outside the ROPE and in the observed direction.

a For each criterion, there was a 1.00 probability of the LOR posterior falling outside the ROPE.

As expected, there was a higher probability that a child would meet any single ASD criterion if they received an ASD diagnosis. This effect was strongest in the social communication criteria (Domain A) because, if ASD diagnosis is provided, all Domain A criteria must be met. As this is not the case for the Domain B (RRBI criteria), there was variability as to how strongly meeting a Domain B criterion predicted overall diagnosis: A diagnosis of ASD was most likely if B2 or B4 was met. Females were more likely to meet Criteria B2 and B4 than were males, but this effect was only weak.

Table 5 presents proportions and relative risks of children in each group for criteria met and partially met. Of note, all but one female demonstrated at least some atypicality in Criterion A3 (difficulties with relationships). Difficulties with relationships may therefore be a key reason that many females present for assessment, regardless of assessment result. Further, males had a higher probability of meeting Criterion B3 (restricted interests; non-ASD males had a 1.7-times higher probability than non-ASD females, and autistic males had a 1.3-times higher probability than autistic females).

**Table 5.** Descriptive statistics and relative risk for criteria met/partially met by sex/gender and assessment result.

Variable		Criterion						
		A1	A2	A3	B1	B2	B3	B4
Males								
Proportion meeting criterion (%)	ASD	100.0	100.0	100.0	68.8	81.8	67.8	89.9
	Non-ASD	51.1	30.0	43.1	30.6	28.2	22.8	28.7
	Relative risk	1.96	**3.33**	2.32	2.25	2.90	2.97	3.13
Proportion with any atypicality (%)	ASD	100.0	100.0	100.0	93.8	93.8	87.6	97.2
	Non-ASD	84.5	69.2	80.7	57.4	71.4	56.4	76.2
	Relative risk	1.18	1.45	1.24	**1.63**	1.31	1.55	1.28
Females								
Proportion meeting criterion (%)	ASD	100.0	100.0	100.0	63.6	85.7	53.9	93.2
	Non-ASD	26.7	29.3	40.4	25.6	32.2	13.3	37.6
	Relative risk	3.75	3.41	2.48	2.48	2.66	**4.05**	2.48
Proportion with any atypicality (%)	ASD	100.0	100.0	100.0	89.0	93.4	83.0	98.1
	Non-ASD	87.7	77.0	92.1	61.2	75.2	34.8	72.7
	Relative risk	1.14	1.30	1.09	1.45	1.24	**2.38**	1.35

ASD: autism spectrum disorder.

Proportion with any atypicality indicates the proportion of children who met or partially met the criterion. All proportions were derived from the logistic regression models. Relative risk was calculated as follows: proportion ASD ÷ proportion non-ASD. The largest relative risk in each set of results is highlighted in boldface.

#### Social communication

For the majority of behaviours examined, the probability that atypicality was reported was higher for children who received an ASD diagnosis. However, there were some exceptions to this, particularly in areas that might be less strongly associated with ASD (e.g. difficulty losing a game). Main effects of assessment result, sex/gender and their interaction on the behaviour and simple main effects (ASD M–F; non-ASD M–F) are provided. Table 6 presents the results of logistic regressions for behaviours with meaningful differences within Criteria A.

**Table 6.** Logistic regression predicting behaviour by assessment result, sex/gender and their interaction: Criteria A.

Behavioural category	Effect of Ax. result		Effect of sex/gender		Ax. result × sex/gender interaction		Prop. diff. M, F (Y–N)	ASD: M–F		Non–ASD: M–F	
	LOR [HDI_80%_]	P_(meaning)_	LOR [HDI_80%_]	P_(meaning)_	LOR [HDI_80%_]	P_(meaning)_		LOR [HDI_80%_]	P_(meaning)_	LOR [HDI_80%_]	P_(meaning)_
Criterion A1											
Parent report											
Sharing interests	**1.24 [0.64, 1.82]**	**1.00**	−0.66 [−1.26, –0.07]	−0.89	1.23 [0.06, 2.40]	0.90	0.23, 0.11	−0.05 [−0.52, 0.43]	−0.44	**–1.28 [−2.34, –0.19]**	**–0.92**
Literal language	−0.13 [−0.60, 0.34]	−0.53	**–0.70 [−1.17, –0.23]**	**–0.95**	0.18 [−0.74, 1.12]	0.54	−0.01, –0.05	−0.60 [−1.11, –0.08]	−0.90	−0.78 [−1.56, –0.00]	−0.87
Diagnostic observations											
Reciprocal conversation	**1.82 [1.36, 2.28]**	**1.00**	0.53 [0.06, 1.00]	0.89	0.42 [−0.50, 1.34]	0.67	0.46, 0.37	**0.74 [0.27, 1.22]**	**0.96**	0.32 [−0.49, 1.10]	0.64
Sharing interests	**2.43 [0.83, 3.97]**	**1.00**	**1.91 [0.35, 3.45]**	**0.97**	−2.97 [−5.92, 0.19]	−0.93	0.09, 0.11	0.43 [−0.19, 1.04]	0.76	0.32 [−0.49, 1.10]	0.64
Content of conversation	**2.31 [1.57, 3.02]**	**1.00**	**1.02 [0.28, 1.74]**	**0.97**	**–2.24 [−3.69, –0.77]**	**–0.99**	**0.28, 0.53**	−0.10 [−0.55, 0.34]	−0.51	**2.16 [0.75, 3.54]**	**0.99**
Teacher report											
Social approach	0.33 [−0.20, 0.83]	0.71	0.50 [−0.03, 1.01]	0.84	0.66 [−0.36, 1.69]	0.76	0.16, 0.00	**0.82 [0.28, 1.37]**	**0.96**	0.16 [−0.70, 1.03]	0.53
Reciprocal conversation	**1.05 [0.49, 1.62]**	**0.99**	**0.87 [0.29, 1.43]**	**0.97**	0.63 [−0.50, 1.76]	0.72	0.33, 0.15	**1.18 [0.61, 1.74]**	**0.99**	0.55 [−0.45, 1.51]	0.73
Criterion A2											
Parent report											
Integration of verbal/NV behaviour	**3.80 [1.93, 5.49]**	**1.00**	**–2.08 [−3.73, –0.42]**	**–0.96**	2.27 [−1.03, 5.50]	0.83	0.14, 0.26	**–0.93 [−1.49, –0.40]**	**–0.98**	**–3.32 [−6.68, –0.14]**	**–0.93**
Facial expression	**1.54 [1.04, 2.04]**	**1.00**	**–1.17 [−1.67, –0.67]**	**–1.00**	0.19 [−0.79, 1.19]	0.54	0.27, 0.34	**–1.07 [−1.52, –0.62]**	**–1.00**	**–1.26 [−2.15, –0.37]**	**–0.95**
Teacher report											
Use of nonverbal comm.	0.65 [0.01, 1.25]	0.89	**0.99 [0.34, 1.60]**	**0.96**	−0.53 [−1.77, 0.72]	−0.68	0.09, 0.15	**0.72 [0.13, 1.27]**	**0.92**	**1.25 [0.13, 2.35]**	**0.92**
Nonverbal understand	**1.30 [0.70, 1.88]**	**1.00**	0.61 [0.02, 1.20]	0.87	1.07 [−0.09, 2.28]	0.85	0.43, 0.17	**1.14 [0.57, 1.73]**	**0.99**	0.07 [−0.94, 1.10]	0.49
Criterion A3											
Parent report											
Submissive/dominating in play	0.28 [−0.13, 0.71]	0.71	**–0.69 [−1.11, –0.25]**	**–0.96**	0.14 [−0.70, 0.99]	0.53	0.08, 0.05	**–0.61 [−1.07, –0.18]**	**–0.93**	−0.76 [−1.46, –0.01]	−0.88
Possessive/losing	**0.80 [0.38, 1.23]**	**0.98**	0.19 [−0.24, 0.62]	0.61	0.79 [−0.07, 1.65]	0.85	0.28, 0.09	**0.58 [0.15, 1.03]**	**0.92**	−0.22 [−0.94, 0.53]	−0.57
Friendship formation	**1.44 [1.01, 1.88]**	**1.00**	−0.07 [−0.51, 0.37]	−0.47	**1.39 [0.53, 2.27]**	**0.97**	**0.48, 0.18**	**0.62 [0.16, 1.08]**	**0.93**	−0.76 [−1.49, –0.00]	−0.87
Diagnostic observations											
Inclusiveness in play	**1.79 [0.95, 2.60]**	**1.00**	0.38 [−0.47, 1.17]	0.67	**–1.85 [−3.46, –0.18]**	**–0.93**	**0.08, 0.22**	−0.55 [−1.11, –0.02]	−0.85	1.29 [−0.23, 2.84]	0.86
Imaginative/spont. play	**1.38 [0.56, 2.17]**	**0.99**	0.06 [−0.74, 0.86]	0.47	**1.88 [0.29, 3.47]**	**0.92**	**0.39, 0.07**	**0.99 [0.39, 1.60]**	**0.97**	−0.89 [−2.32, 0.63]	−0.76
Teacher report											
Friendship formation	**0.99 [0.44, 1.51]**	**0.99**	0.55 [0.01, 1.09]	0.86	0.43 [−0.63, 1.52]	0.65	0.29, 0.17	**0.76 [0.21, 1.33]**	**0.94**	0.34 [−0.59, 1.24]	0.63

ASD: autism spectrum disorder; Ax.: Assessment; LOR: log odds ratio; HDI: highest density interval; NV: nonverbal.

Criterion A1: deficits in social-emotional reciprocity; A2: deficits in nonverbal communication behaviours; A3: deficits in developing, maintaining and understanding relationships (DSM-5). Positive LORs (ASD assessment result) = greater probability of being reported if the assessment result was positive for ASD; positive LORs (sex/gender) = greater probability of being reported for males. Differences in boldface indicate the HDI_80%_ lay entirely outside the ROPE. P_(meaningful)_ indicates the probability that the true difference fell outside the ROPE and in the observed direction. Prop. Diff. = proportion of children with behaviour reported for males (Yes–No ASD result) and females (Yes–No ASD result).

In most cases where there was evidence of an interaction between sex/gender and assessment result (five of the behaviours assessed; P_(meaningful)_ > .85), that interaction was due to a large sex/gender difference for one level of diagnosis (i.e. ASD or non-ASD) accompanied by a negligible difference for the other level of diagnostic outcome.

As expected, behaviours examined were differentially predictive of ASD diagnosis (effect of assessment result). However, sex/gender meaningfully predicted atypicality reported in many areas (e.g. *facial expression*, parent-reported atypicality more likely for females; *sharing interests*, diagnostician observed atypicality more likely for males). Some atypicality was less strongly predictive of diagnosis for females (e.g. *friendship formation*, parent report) and more predictive for other behaviours (e.g. *content of conversation*, diagnostician observation).

#### Repetitive and restricted behaviours and interests

Table 7 presents the meaningful results of logistic regressions for RRBIs. There was strong evidence of an effect of sex/gender for many behaviours, including specific speech stereotypies (e.g. *repetitive speech*, greater likelihood in males; *neologisms*, greater likelihood in females) and within other RRBI criteria (e.g. *visual avoiding* behaviours, greater likelihood of parent-reported atypicality in females; *restricted interests*, greater likelihood of diagnostician observed atypicality in males). Specific parent-reported interests were also more common in either males or females. The presence of some behaviours was more predictive of ASD diagnosis for females (e.g. *odd prosody*) and others for males (e.g. parent-reported *distress at change*).

**Table 7.** Logistic regression predicting behaviour by assessment result, sex/gender and their interaction: Criteria B.

Behavioural category	Effect of Ax. result		Effect of sex/gender		Ax. result × sex/gender interaction		Prop. diff. M, F (Y–N)	ASD: M–F		Non-ASD: M–F	
	LOR [HDI_80%_]	P_(meaning)_	LOR [HDI_80%_]	P_(meaningful)_	LOR [HDI_80%_]	P_(meaning)_		LOR [HDI_80%_]	P_(meaning)_	LOR [HDI_80%_]	P_(meaning)_
Criterion B1											
Parent report											
Motor stereotypies	1.10 [0.58, 1.61]	**1.00**	0.34 [−0.19, 0.84]	0.73	−0.82 [−1.82, 0.24]	−0.82	0.15, 0.28	−0.07 [−0.51, 0.38]	−0.47	0.73 [−0.20, 1.65]	0.82
Toe walking	0.79 [−0.01, 1.54]	.90	0.76 [−0.03, 1.53]	0.89	**–1.96 [−3.48, –0.38]**	**–0.96**	**–0.03, 0.15**	−0.22 [−0.77, 0.36]	−0.61	**1.74 [0.27, 3.15]**	**0.96**
Speech/language	**3.38 [1.95, 4.72]**	**1.00**	**2.28 [0.87, 3.63]**	**1.00**	**–3.63 [−6.29, –0.80]**	**–0.99**	**0.34, 0.44**	0.47 [0.02, 0.91]	0.86	**4.05 [1.26, 6.74]**	**1.00**
Echolalia	**0.97 [0.11, 1.75]**	**.94**	**0.95 [0.12, 1.75]**	**0.93**	−0.81 [−2.42, 0.81]	−0.72	0.07, 0.08	0.54 [−0.06, 1.13]	0.83	1.33 [−0.19, 2.84]	0.88
Third-person referencing	0.15 [−0.87, 1.15]	.53	−0.67 [−1.66, 0.34]	−0.78	**1.77 [0.26, 3.67]**	**0.87**	**0.02, –0.04**	0.19 [−0.86, 1.20]	0.54	−1.55 [−3.26, 0.11]	−0.88
Neologisms	0.43 [−0.10, 0.97]	.80	**–0.85 [−1.39, –0.31]**	**–0.97**	**1.37 [0.28, 2.42]**	**0.94**	**0.13, –0.05**	−0.17 [−0.68, 0.35]	−0.57	**–1.54 [−2.46, –0.59]**	**–0.98**
Repetitive speech	**0.99 [0.47, 1.49]**	**.99**	**0.99 [0.47, 1.49]**	**0.99**	−0.97 [−1.99, 0.05]	−0.87	0.12, 0.27	0.50 [0.05, 0.93]	0.88	**1.47 [0.55, 2.38]**	**0.98**
Accents	**1.14 [0.43, 1.84]**	**.98**	**–1.25 [−1.94, –0.53]**	**–0.98**	0.29 [−1.11, 1.69]	0.57	0.09, 0.15	**–1.09 [−1.67, –0.54]**	**–0.99**	**–1.39 [−2.67, –0.12]**	**–0.91**
Unusual noises	**0.98 [0.49, 1.47]**	**.99**	0.38 [−0.11, 0.89]	0.77	0.73 [−0.23, 1.73]	0.79	0.28, 0.11	**0.74 [0.30, 1.21]**	**0.97**	0.02 [−0.89, 0.87]	0.45
Talking to self	−0.17 [−1.29, 0.87]	−.53	**–1.35 [−2.42, –0.23]**	**–0.94**	0.68 [−1.40, 2.82]	0.64	0.00, –0.03	−0.98 [−2.23, 0.20]	−0.84	−1.69 [−3.42, 0.04]	−0.90
Odd prosody	**1.24 [0.51, 1.97]**	**.99**	**1.12 [0.36, 1.82]**	**.98**	**–2.75 [−4.15, –1.26]**	**–1.00**	**–0.03, 0.33**	−0.25 [−0.73, 0.20]	−0.66	**2.50 [1.10, 3.87]**	**1.00**
Object use	1.36 [0.72, 1.99]	**1.00**	0.07 [−0.57, 0.69]	0.48	−0.71 [−1.95, 0.56]	−0.74	0.18, 0.24	−0.28 [−0.76, 0.20]	−0.69	0.42 [−0.72, 1.59]	0.64
Deconstruct-ion	**3.22 [1.45, 4.86]**	**1.00**	−0.78 [−2.38, 0.81]	−0.72	**4.11 [0.88, 7.20]**	**0.97**	**0.24, 0.05**	−0.39 [−1.02, 0.23]	−0.73	−0.83 [−2.03, 0.36]	−0.79
Teacher report											
Stereotypical movement	0.43 [−0.31, 1.15]	.72	0.61 [−0.14, 1.34]	0.82	1.05 [−0.42, 2.51]	0.79	0.14, 0.00	**1.12 [0.33, 1.88]**	**0.96**	0.08 [−1.16, 1.33]	0.49
Stereotypical speech/lang.	**1.02 [0.17, 1.81]**	**.95**	**1.20 [0.37, 2.03]**	**0.97**	−0.35 [−1.97, 1.31]	−0.58	0.18, 0.12	**1.01 [0.35, 1.68]**	**0.96**	**1.36 [−0.19, 2.80]**	**0.89**
Stereotypical object use	−0.92 [−2.65, 0.79]	−.74	**2.49 [0.64, 4.31]**	**0.98**	**4.34 [1.09, 7.72]**	**0.97**	0.10, –0.04	**4.54 [1.66, 7.35]**	**1.00**	0.29 [−1.62, 2.13]	0.55
Criterion B2											
Parent report											
Distress at change	**1.33 [0.89, 1.75]**	**1.00**	−0.37 [−0.81, 0.07]	−0.79	**1.34 [0.48, 2.21]**	**0.97**	**0.46, 0.16**	0.30 [−0.17, 0.76]	0.70	**–1.04 [−1.78, –0.32]**	**–0.95**
Routine adherence	**1.04 [0.60, 1.47]**	**1.00**	**–0.61 [−1.05, –0.16]**	**–0.93**	0.83 [−0.04, 1.71]	0.86	0.31, 0.15	−0.19 [−0.62, 0.25]	−0.61	**–1.03 [−1.79, –0.26]**	**–0.94**
Cognitive rigidity	**1.82 [1.36, 2.27]**	**1.00**	−0.21 [−0.66, 0.26]	−0.62	**1.12 [0.23, 2.04]**	**0.93**	**0.53, 0.30**	0.35 [−0.13, 0.80]	0.75	−0.77 [−1.55, 0.00]	−0.86
Diagnostic observations											
Cognitive rigidity	**1.31 [0.61, 2.00]**	**.99**	−0.48 [−1.18, 0.22]	−0.76	−0.11 [−1.49, 1.27]	−0.51	0.11, 0.17	−0.54 [−1.07, –0.01]	−0.86	−0.43 [−1.74, 0.82]	−0.63
Criterion B3											
Parent report											
Programme/character	0.18 [−0.27, 0.62]	.60	−0.03 [−0.48, 0.43]	−0.42	**–1.25 [−2.17, –0.36]**	−0.95	−0.01, 0.18	**–0.65 [−1.10, –0.18]**	**–0.94**	0.59 [−0.20, 1.36]	0.80
Random objects	**0.72 [0.27, 1.17]**	**.97**	−0.44 [−0.91, –0.00]	−0.84	**1.43 [0.56, 2.33]**	**0.97**	**0.29, 0.00**	0.27 [−0.18, 0.70]	0.69	**–1.16 [−1.92, –0.37]**	**–0.96**
Vehicles	−0.90 [−1.67, –0.02]	−.90	**2.17 [1.31, 2.99]**	**1.00**	**3.38 [1.73, 5.02]**	**1.00**	**0.15, –0.12**	**3.77 [2.41, 5.00]**	**1.00**	0.46 [−0.51, 1.46]	0.68
Screens	**1.49 [0.73, 2.27]**	**1.00**	**1.96 [1.15, 2.73]**	**1.00**	−0.61 [−2.07, 0.97]	−0.67	0.27, 0.14	**1.65 [1.11, 2.16]**	**1.00**	**2.25 [0.82, 3.70]**	**0.99**
Craft	0.72 [0.07, 1.38]	.90	**–0.94 [−1.59, –0.28]**	**–0.95**	−0.09 [−1.40, 1.19]	−0.50	0.05, 0.11	**–0.97 [−1.58, –0.35]**	**–0.97**	−0.88 [−2.06, 0.24]	−0.81
People	**2.83 [0.94, 4.67]**	**.99**	**–2.18 [−3.90, –0.42]**	**–0.96**	2.43 [−0.99, 5.76]	0.84	0.05, 0.09	**–0.94 [−1.79, –0.10]**	**–0.91**	−3.26 [−6.50, 0.07]	−0.92
Diagnostic observations											
Restricted interests	**3.14 [1.62, 4.57]**	**1.00**	**2.18 [0.66, 3.60]**	**1.00**	−3.01 [−5.86, –0.06]	−0.95	0.12, 0.15	**0.67 [0.19, 1.13]**	**0.94**	**3.63 [0.74, 6.45]**	**0.98**
Teacher report											
Restricted interests	**1.19 [0.55, 1.83]**	**.99**	**1.05 [0.40, 1.69]**	**0.98**	0.41 [−0.85, 1.69]	0.63	0.33, 0.16	**1.25 [0.65, 1.83]**	**0.99**	0.83 [−0.33, 1.93]	0.81
Criterion B4											
Parent report											
Oral: avoiding	**1.04 [0.52, 1.54]**	**.99**	−0.33 [−0.84, 0.19]	−0.71	**1.35 [0.32, 2.36]**	**0.94**	**0.29, 0.07**	0.34 [−0.10, 0.82]	0.75	−0.99 [−1.93, –0.10]	−0.89
Visual: seeking	**0.71 [0.19, 1.25]**	**.94**	−0.61 [−1.14, –0.08]	−0.89	0.82 [−0.22, 1.88]	0.81	0.15, 0.06	−0.20 [−0.69, 0.29]	−0.73	**–1.02 [−1.97, –0.10]**	**–0.90**
Visual: avoiding	**0.96 [0.11, 1.77]**	**.93**	**–1.05 [−1.86, –0.21]**	**–0.95**	**2.88 [1.21, 4.45]**	**0.99**	**0.14, –0.06**	0.39 [−0.23, 1.01]	0.73	**–2.47 [−4.00, –0.99]**	**–0.99**

ASD: autism spectrum disorder; Ax.: Assessment; LOR: log odds ratio; HDI: highest density interval.

Criterion B1: stereotyped/repetitive motor movements, use of objects or speech; B2: insistence on sameness, routines or ritualised behaviour; B3: restricted and fixated interests; B4: hyper-/hypo-reactivity to sensory input (DSM-5). Positive LORs (ASD assessment result) = greater probability of being reported if the assessment result was positive for ASD; positive LORs (sex/gender) = greater probability of being reported for males. Differences in boldface indicate the HDI80% lay entirely outside the ROPE. P(meaningful) indicates the probability that the true difference fell outside the ROPE and in the observed direction. Prop. Diff. = proportion of children with behaviour reported for males (Yes–No ASD result) and females (Yes–No ASD result).

## Discussion

The present study examined differences between male and female children who were referred for and undertook a formal autism assessment. Both ASD and non-ASD females were less likely than their male counterparts to meet Criterion B3 (restricted interests). Furthermore, there was strong evidence that their restricted interests differed to those of males. Non-ASD (subclinical) females were found to lack sufficient atypicality for diagnosis in some domains (particularly Criterion A1, social-emotional reciprocity). Some ASD behaviours were more likely to be reported for females than for males (e.g. atypicality in facial expression), and notably, there were some behaviours diagnostic of ASD and often seen in males which were not commonly observed in females (e.g. difficulties with imaginative play). Finally, other behaviours were found to be more *diagnostic* (i.e. more strongly associated with assessment result) for males (e.g. repetitive deconstruction of objects) and others for females (e.g. speech/language mannerisms). Together, these findings may assist in understanding why females may be less likely to be diagnosed with ASD than males and provide direction for future assessment protocols.

### Sex/gender differences in ASD presentation

Consistent with the theoretical framework outlined by Lai et al. (2015), evidence of sex/gender differences in the present study was most apparent at the level of specific behaviours. Parents of females were meaningfully more likely to report difficulty in some behaviours (e.g. difficulties with eye contact, literal interpretation of language) and in interests and specific stereotypical mannerisms (e.g. echolalia, toe-walking). Surprisingly, parents of females were more likely to report atypicality in several behaviours within nonverbal behaviour (A2) than parents of males (e.g. decreased integration of verbal and nonverbal communication, reduced or increased use of facial expressions), but despite this, these behaviours did not translate to a higher probability that females would meet this criterion during the diagnostic assessment.

Broadly, the above identified sex/gender differences in social behaviours are consistent with prior investigations (e.g. Bitsika & Sharpley, 2019; Hiller et al., 2014). Such social differences may be the result of higher parental expectations of their daughters’ social abilities affecting their reporting during assessment. Furthermore, these differences may reflect social camouflaging or higher levels of social motivation among girls (Lockwood Estrin et al., 2021) leading to active concealment of traits that are viewed as unusual by peers (Hull et al., 2017). It is also likely that different levels of social skills are expected of girls compared to boys, and thus certain difficulties, considered more atypical, may need to be present before a referral or ASD diagnosis is made (Dworzynski et al., 2012; Tsirgiotis et al., 2022a).

Females were significantly less likely than males to meet Criterion B3 and demonstrate restricted interests. As also reported by Hiller et al. (2014) and McFayden et al. (2019), sex/gender differences were present in the nature of restricted interests, with males more likely to present with obsessions with vehicles and screens, and females more likely to be interested in other people and craft activities. A novel finding of the present study is that some interests were more strongly associated with ASD diagnosis for males (e.g. random objects or people) or females (e.g. animals or specific programmes/characters). As previously suggested (e.g. Nowell et al., 2019), restricted interests that are less overtly atypical in orientation may be diagnostically overlooked.

Furthermore, the presence of certain sensory avoiding behaviours was more diagnostic for males than for females although parents of females were also more likely than parents of males to report them. Findings surrounding sensory sex/gender differences have been inconsistent, both broadly (Frazier & Hardan, 2017; Lai et al., 2011) and in specific sensory sensitivities (Bitsika et al., 2018). As females were found to be slightly more likely to meet Criterion B4 (sensory sensitivities) in the present study, further investigation into whether elevated sensory difficulties constitute an important element of the female presentation is warranted.

In addition to different restricted interests and sensory sensitivities, males and females also presented with different parent-reported stereotypical behaviours. In contrast with other findings (Antezana et al., 2019; Tsirgiotis et al., 2022b), this was least apparent in stereotypical body use behaviours, with the only meaningful difference being a higher probability of repetitive body use mannerisms (such as hair twirling or nail biting) among females. In contrast, there was meaningful evidence of sex-/gender-specific profiles of speech and language mannerisms. Males had a higher probability of presenting with parent-reported speech mannerisms in general, and repetitive speech and echolalia specifically, while females were more likely to present with neologisms, unusual accent use, and talking to oneself. Sex/gender differences in speech and language use have remained largely overlooked in the literature. While it is unknown why stereotypical speech profiles may differ by sex/gender, this may result from differences in language development or as a consequence of camouflaging and imitation (e.g. accents may be imitated from others) and is worthy of further exploration.

Although not a specific focus of this article, levels of atypicality reported differed according to the source, suggesting that the presentation of autistic females may vary with environment. Unlike in parent report (similar probability of reported atypicality between males and females), teachers were more likely to report atypicality for males (see also Mandy et al., 2011; Posserud et al., 2006). There was also weak evidence for this for diagnostician observations. A less atypical behaviour noted by diagnosticians from clinical observations gives cause for caution when relying on observation schedules such as the Autism Diagnostic Observation Schedule second edition (ADOS-2) (Adamou et al., 2018; Lai et al., 2011). The discrepancies across reporting sources suggest that there may be differences in a female’s ASD presentation according to her environment and lends support to the camouflage hypothesis; that is, females may be more highly motivated or better able to conceal and compensate for ASD-related difficulties in some environments (Hull et al., 2017; Rynkiewicz et al., 2016). Alternatively, the asymmetry in reporting for males and females may reflect bias in interpretation of behaviours (discussed in following sections).

This study also corroborates previous research suggesting that females may come to clinical attention later in the developmental period than males (Begeer et al., 2013; Mandy et al., 2018). This may reflect a genuinely later onset of ASD characteristics (especially social difficulties), but more likely the increasing overtness of difficulties with increased social demands, coupled with other factors which delay referral.

### Females under the diagnostic threshold

In addition to presenting with different stereotypic behaviours and restricted interests to males, females were less likely to meet Criteria B1 and B3 than their male counterparts, regardless of diagnostic result. In some instances, the stereotypical behaviours that were more common in females were less strongly associated with ASD diagnosis than those typically presented by males. Indeed, repetitive body use and neologisms were more common among non-ASD females than in any other group, and hand-mannerisms, rocking/jumping and repetitive play were as common in non-ASD females as in children with ASD, irrespective of sex/gender. This suggests that sex-/gender-specific profiles of stereotypical behaviours may exist and that those presented by females may not perfectly coincide with ASD conceptualisation (see also Lai et al., 2015; Van Wijngaarden-Cremers et al., 2014). It is possible, therefore, that these sex-/gender-specific profiles may contribute to underdetection of ASD in females.

As expected, non-ASD females lacked a number of difficulties that were present among ASD females. In particular, non-ASD females lacked sufficient difficulty in social-emotional reciprocity (Criterion A1; where non-ASD males were approximately half as likely to partially meet and twice as likely to fully meet the criterion as non-ASD females). Two possible reasons for this can be deduced from this study: first, a number of parent-reported A1 difficulties were more strongly associated with diagnosis for males, and second, males were more likely to display a number of difficulties in this area during assessment. Importantly, evidence suggested that non-ASD females were more likely to have parent-reported atypicality than non-ASD males in the social communication and RRBI domain, whereas for diagnostician observation and teacher report, non-ASD males were weakly more likely to report atypicality. Together, these findings suggest that, for subclinical females with autistic traits, insufficient evidence of difficulty observed during assessment and/or in the school environment may contribute to why an ASD diagnosis may not be made.

### Sex/gender differences in behaviours associated with diagnosis

Many behaviours were more *diagnostic* for males and others for females – that is, their presence was more strongly predictive of ASD diagnosis for males or females. There was unequivocal evidence of such an interaction for 18 behaviours. A minority of these (22.2%) were found in the social communication domain, with the majority in the RRBI domain and within stereotypic behaviours and restricted interests in particular. The presence of interactions in the RRBI domain is consistent with the findings of Duvekot et al. (2017), wherein RRBI difficulties were more strongly associated with ASD diagnosis for males than for females. The present study builds on this finding, identifying specific behaviours across all domains, which may contribute unevenly to ASD diagnosis for males and females.

This evidence may be interpreted in two main ways. First, diagnosticians may have a different view of which behaviours are most diagnostic for males and females, suggesting they have an implicit understanding of differing ASD presentations. Second, diagnosticians may interpret behaviours differently depending on the child’s sex/gender. The latter is not necessarily evidence of bias, as this could also reflect differences in the typical developmental trajectories of males and females. Differences in the interpretation of behaviours associated with ASD reinforce the need for sex-/gender-specific psychometric assessment tools, or at least norms, such that the behaviours are compared with neurotypical behaviours and social expectations specific to that sex/gender (Lai & Baron-Cohen, 2015; Lai et al., 2022).

In the absence of biomarkers, ASD diagnosis is made clinically at assessment. The assessment process relies on reviewing and compiling evidence of an individual’s development to determine whether there is enough atypicality with sufficient functional impact in a variety of ASD-defining domains to warrant diagnosis (American Psychiatric Association, 2013). This means enforcing a binary decision (providing an ASD diagnosis or not) upon a series of continua, each with a degree of associated subjectivity and personal biases (e.g. parental description of behaviours and functional impact, diagnosticians’ threshold for atypicality). Coupled with the less ‘classic’ ASD presentations of many females, this means diagnosticians may reasonably lack confidence in their assessment (Tsirgiotis et al., 2022a). Thus, we suggest the following to diagnosticians (see also; Cumin et al., 2022). First, employ measures with female norms (e.g. Social Responsiveness Scale; Constantino, 2011), many of which are emerging, and actively assess for camouflaging (assessing behaviours in many settings, using measures such as the Camouflaging Autistic Traits Questionnaire; Hull et al., 2019; and allowing ample time for rapport building so that camouflaging efforts may relax). Second, adapt the parent and teacher interview to carefully examine female manifestations of ASD behaviours (e.g. less atypical repetitive mannerisms). Third, if the diagnostic outcome is uncertain, discuss the case with a peer and conduct a clinical review after a period of time.

Accurate identification of ASD is essential in providing clarity and validity to the experiences of individuals and families, to identify difficulties that require support to assist in accessing such support, and to add a framework with which to understand and celebrate ASD traits (Bargiela et al., 2016). We suggest that careful assessment of functional impact and whether ASD-specific support may be beneficial may be the keys in determining the appropriateness of an ASD diagnosis for children whose presentations lie narrowly outside the criteria or present less classically.

### Strengths and limitations

A particular strength of this study was the bottom-up approach to data collection. This enabled movement beyond existing psychometric instruments, which may perpetuate traditional views of ASD presentation and lack sensitivity to the difficulties of females (see Hiller et al., 2014; Lombardo et al., 2019). Another strength was the inclusion of children under the diagnostic threshold (i.e. who were formally assessed and received a negative ASD result), which allowed for the investigation of the sex/gender differences in the ASD features that are commonly absent and therefore reasons that ASD diagnoses are not made. Examination of a large array of ASD behaviours as reported by several different sources allowed for detailed study of sex/gender differences in ASD presentation.

However, several limitations restrict the conclusions that can be made. First, data were collected from a single private clinic and from the assessment reports of one group of diagnosticians (*n* = 7). Thus, the extent to which these results are generalisable is unknown. Second, while a primary contribution of this study was the investigation of the presentations of females who have many ASD traits but are not diagnosed, it only included children who presented for specialist assessment. As a result, a significant subgroup of children with many ASD traits, or who may meet diagnostic criteria if they came to clinical attention, were not included. This is likely to be a disproportionate issue for females. Because, for these individuals, their ASD traits have been overlooked, normalised or otherwise explained, it may be challenging for research to identify and explore their experiences. Finally, given many behaviours were coded as a binary variable (i.e. significant atypicality reported or not), it may be of interest to examine the degree to which each difficulty presents for males and females, as well as the functional limitations that difficulty poses for the individual, either in isolation or in combination with other difficulties. Exploring these differences may help to further clarify the female ASD presentation and the clinical significance of difficulties of females under the diagnostic threshold.

### Conclusions and future directions

In this study, a large number of sex/gender differences were found in the probability of presenting with particular autistic behaviours, as reported by parents and teachers and observed by diagnosticians during assessment. Females were substantially less likely to meet Criterion B3 (restricted interests), and this was especially the case for non-ASD (subclinical) females. There was evidence that the nature of restricted interests and stereotypical behaviour profiles differed by sex/gender, and for females, many of these behaviours were less strongly associated with ASD diagnosis than those presented by males. Non-ASD females also lacked a number of social-emotional reciprocity difficulties. In addition, there was a meaningful contrast in the levels of atypicality reported by parents and the observations of diagnosticians and teachers for females, suggesting that camouflage in social environments, differences in the overtness of difficulties or sex-/gender-based interpretation biases may contribute to a negative ASD result or at least require greater impairment in other areas in order to qualify for diagnosis. Finally, for a number of behaviours, their presence was more indicative of a positive diagnosis for males than for females or vice versa, suggesting gender differences in how atypical behaviours are perceived. Given these results, it may be necessary to frame diagnostic criteria more flexibly so as not to exclude females who do not present with certain difficulties or for whom less atypicality is identified in social environments.

## Supplemental Material

sj-docx-1-aut-10.1177_13623613231190682 – Supplemental material for A comparison of the presentations of males and females with autism spectrum disorder and those narrowly below the diagnostic threshold Supplemental material, sj-docx-1-aut-10.1177_13623613231190682 for A comparison of the presentations of males and females with autism spectrum disorder and those narrowly below the diagnostic threshold by Joanna M Tsirgiotis, Robyn L Young and Nathan Weber in Autism

## Notes

1. We use the term sex/gender to acknowledge the overlap between biological characteristics (sex) and socially constructed attributes and expectations (gender) as proposed by Springer et al. (2011).

2. An attempt was made to statistically control for intellectual ability in the initial analyses. However, this was not practical given that IQ data were only available for *n* = 143 participants (64.4%). Children with intellectual disabilities were, therefore, excluded.

3. The teacher questionnaire is provided in the Supplementary material.

4. Total scores on these tools were collected and analysed, but given the variety of assessment tools used, there were insufficient observations to make meaningful conclusions. Therefore, these results are not presented.

5. For a description of the operationalisation of each behaviour, refer to the Supplementary material.

6. Initially, the outcome variables (ASD behaviours) were coded in tertiary form, that is, (0) *no ASD-related atypicality*, (1) *subclinical atypicality/partially indicated* or (2) *atypicality present*. However, due to reliability concerns and the relatively restricted sample size, these were collapsed to binary variables, with (1) *subclinical atypicality/partially indicated* merged with (0) *no ASD-related atypicality*. These levels were collapsed to preserve the comparator of ‘enough atypicality to contribute to a met criterion’ versus ‘insufficient atypicality’.

7. These data are presented in graphic form in the Supplementary material.
