# Supplementary material for: A comparison of the presentations of males and females with autism spectrum disorder and those narrowly below the diagnostic threshold
Source: Autism. 2023 Aug 22;28(4):1029–44. doi: 10.1177/13623613231190682 (PMC10981200; doi:10.1177/13623613231190682)
Supplement: sj-docx-1-aut-10.1177_13623613231190682 – Supplemental material for A comparison of the presentations of males and females with autism spectrum disorder and those narrowly below the diagnostic threshold [file sj-docx-1-aut-10.1177_13623613231190682.docx]

**Supplementary material**

**Supplementary Form 1**

*Teacher Questionnaire*

Child's name

How long have you known this child?

Year level of child

Your name, name of school

1. Briefly describe this child's learning ability/academic progress. Do you have any academic or cognitive concerns?
2. How would you describe the child’s social approaches?
3. Does the child show, bring, or point out objects of interest? Do they share events/items they are proud of? Will they share information regarding home or weekend events?
4. Describe their ability to share, take turns, wait, and ask for help. Are they possessive of any items?
5. How would you describe your student’s ability to engage in reciprocal communication/conversation?
6. How would you describe their empathy? How do they react or respond when others are upset/hurt? Do they shown concern or comfort and how do they show it?
7. What does the student do when they are upset/hurt?
8. Describe the child’s use of eye contact.
9. Does the child use gestures and body language to convey their needs, express feelings or describe events?
10. Does the child express varied facial expressions?
11. Can they read and understand others non-verbal communication (e.g., their body language and facial expressions). Do they respond to such cues and adjust their behaviour?
12. Describe the child’s friendship group and play with peers.
13. How do they participate in group work or activities? If there is conflict with peers, how do they deal with it?
14. Describe their relationship and connection with teachers and other adults. Do they adjust their behaviour between peers and adults? Are they aware of social hierarchies?
15. Does the child demonstrate any odd or repetitive movements of their body?
16. Does the child demonstrate any unusual qualities to their speech?
17. Does the child do anything unusual with objects or focus in on parts of objects?
18. Do they have any difficulties with change and how do they cope with change?
19. Do they need to follow any specific/pedantic rituals?
20. Do they have any preoccupations with certain objects, activities or topics?
21. Have they developed any strong attachments to or preoccupation with objects?
22. Do they demonstrate any sensory based behaviours such as:
23. Sensitivity to any sounds or general background noise
24. Seeks/avoids touch
25. Unusual smelling
26. Oral
27. Visual behaviours
28. Apparent indifference to temperature
29. Pain threshold

Other comments/concerns:

**Supplementary Table 1**

*Operationalisation of Behaviours Examined*

| Criterion: Source | Behavioural category | Operationalisation |
| --- | --- | --- |
| **Criterion A1** | ***Deficits in socio-emotional reciprocity*** | **Present = 2, some/partially indicated= 1, not present= 0 for each group of behaviours listed below** |
| Parent report | Social approach | Atypical initiation, response, appearing ‘in own world, inappropriate timing of approach, approaching strangers |
|  | Social norms | Lack of awareness of boundaries/personal space, social manners, socially inappropriate comments |
|  | Reciprocal conversation | Difficulties with conversation reciprocity, e.g., turn taking, asking questions, interrupting |
|  | Sharing interests | Difficulty sharing in a topic of conversation, joint attention |
|  | Sharing emotion | Difficulty sharing in others’ emotions, reciprocal smiling, shared enjoyment |
|  | Conversation content | Unusual conversation features, e.g., excessive detail, monologing, redirecting topic |
|  | Literal language | Literal interpretation of jokes/sarcasm |
| Diagnostic observation | As above | As above |
| **Criterion A2** | ***Deficits in nonverbal communicative behaviours used for social interaction*** | **Present = 2, some/partially indicated= 1, not present= 0 for each group of behaviours listed below** |
| Parent report | Integration of verbal/nonverbal behaviour | Difficulties with consistency between nonverbal and verbal behaviour (e.g., smiling when sounding angry) |
|  | Eye contact | Atypicality including absence, staring, inconsistency |
|  | Use of nonverbal communication | Atypicality including exaggerated or absent descriptive/social/emphatic gestures |
|  | Facial expression | Difficulty including exaggerated, absent, or ill-suited to context |
|  | Nonverbal understanding | Difficulties in recognising/understanding nonverbal communication |
|  | Response to nonverbal behaviour | Difficulty in spontaneity, regularity and appropriateness of response to non-vernal behaviour |
|  | Emotional regulation | Difficulty regulating emotions, comfort seeking |
| Diagnostic observation | Integration of verbal/nonverbal behaviour | Difficulties with consistency between nonverbal and verbal behaviour (e.g., smiling when sounding angry) |
|  | Eye contact | Atypicality including absence, staring, inconsistency |
|  | Use of nonverbal communication | Atypicality including exaggerated or absent descriptive/social/emphatic gestures |
|  | Facial expression | Difficulty including exaggerated, absent, or ill-suited to context |
|  | Nonverbal understanding | Difficulties in recognising/understanding nonverbal communication |
| **Criterion A3** | ***Deficits in developing and maintaining relationships appropriate to developmental level*** | **Present = 2, some/partially indicated= 1, not present= 0 for each group of behaviours listed below** |
| Parent report | Adjusting behaviour for situation | Difficulty modifying behaviour to environment or the person with whom the child is interacting, understanding of social hierarchies |
|  | Imagination/spontaneity in play | Difficulty displaying novel or imaginative play themes, use of play scripts |
|  | Submissive/dominating in play | Tendency to allow others to direct the child in play, or to impose their own play ideas upon others |
|  | Possessive/difficulty losing | Possessiveness of objects, difficulty losing a game |
|  | Friendship formation | Difficulty making friends, initiating a relationship |
|  | Friendship maintenance | Difficulty maintaining friendships, resolving conflict |
|  | Social motivation | Absent or excessive motivation for friendships or social interaction |
|  | Consistent companions | Lack of consistent companions, superficial companionship (e.g., parallel play) |
| Diagnostic observation | Friendship understanding | Difficulty understanding what a friend means, how to be a friend, interests of friends etc. |
|  | Inclusiveness of assessor in play | Degree to which the child excluded the assessor in play, and appropriateness of play behaviour (e.g., submissive or dominating |
|  | Imagination, spontaneity in play | Difficulty displaying novel or imaginative play themes, use of play scripts. For older children, engagement in non-clinical discussion or structured play (e.g., board games) |
| **Criterion B1** | ***Stereotyped or repetitive speech, motor movement or use of objects*** | **Present = 2, some/partially indicated= 1, not present= 0 for each group of behaviours listed below** |
| Parent report | Stereotypical movement | Overall level of ASD-consistent atypicality regarding stereotypical movement: pervasiveness, variety, frequency, and functional disruption of behaviours |
|  | Specific motor behaviours | Specify (i.e., list number corresponding to the behaviour):   1. Toe walking 2. Flapping 3. Spinning 4. Gross motor mannerism (e.g., unusual gait) 5. Rocking/swaying/jumping 6. Rigidity (physical) 7. Hand mannerisms (e.g., twinkling or posturing) 8. Mouth mannerisms (e.g., grimacing) 9. Self-injurious (e.g., head banging) 10. Repetitive body use (e.g., thumb sucking, picking at skin) |
|  | Stereotypical speech/ language | Overall level of ASD-consistent atypicality regarding stereotypical speech: pervasiveness, variety, frequency, and functional disruption of behaviours |
|  | Speech/language | Specify (i.e., list number corresponding to the behaviour):   1. Echolalia 2. Third person referencing 3. Neologisms/idiosyncratic speech 4. Pronoun reversal 5. Repetitive speech 6. Accents 7. Unusual noises, self-induced noises 8. Talking to self 9. Odd prosody/tone/volume |
|  | Stereotypical object use | Overall level of ASD-consistent atypicality regarding stereotypical object use: pervasiveness, variety, frequency, and functional disruption of behaviours |
|  | Object use | Specify (i.e., list number corresponding to the behaviour):   1. Lining up 2. Grouping 3. Spinning/flicking/pushing 4. Repetitive play/ object use 5. Deconstructing/attention to parts of objects |
| Diagnostic observation | Stereotypical movement | Overall level of ASD-consistent atypicality regarding stereotypical movement: pervasiveness, variety, frequency, and functional disruption of behaviours |
|  | Stereotypical speech | Overall level of ASD-consistent atypicality regarding stereotypical speech: pervasiveness, variety, frequency, and functional disruption of behaviours |
|  | Stereotypical object use | Overall level of ASD-consistent atypicality regarding stereotypical object use: pervasiveness, variety, frequency, and functional disruption of behaviours |
| **Criterion B2** | ***Excessive adherence to routines, ritualised patterns of verbal or nonverbal behaviour, or excessive resistance to change*** | **Present = 2, some/partially indicated= 1, not present= 0 for each group of behaviours listed below** |
| Parent report | Distress at change | Distress associated with novelty, change or unfamiliarity |
|  | Routine adherence | Insistence on unusual or pedantic rituals that are not necessarily functional |
|  | Task switching/transitioning | Difficulty switching tasks or transitioning between activities, need for completion |
|  | Cognitive rigidity | Black and white thinking, rule adherence |
| Diagnostic observation | Difficulties with transitioning | Difficulty switching tasks or transitioning between activities, need for completion |
|  | Cognitive rigidity | Black and white thinking, rule adherence |
|  | Routine adherence | Development of unusual or pedantic rituals that are not necessarily functional |
| **Criterion B3** | ***Highly restricted, fixated interests that are abnormal in intensity or focus.*** | **Present = 2, some/partially indicated= 1, not present= 0 for each group of behaviours listed below** |
| Parent report | Restricted interests | Specify (i.e., list number corresponding to the behaviour):   1. Specific program/character 2. Random objects (e.g., rocks, shells) 3. Vehicles (including toy vehicles) 4. Toys (not included above e.g., teddy bear) 5. Screens (e.g., video games) 6. Animals 7. Systems (e.g., numbers, routes, schedules) 8. Craft/art (e.g., drawing) 9. Sport/activity (e.g., sailing) 10. People (e.g., celebrities or someone known to the child) |
| Diagnostic observation | Restricted interests | Degree to which a restricted interest was apparent during assessment |
| **Criterion B4** | ***Hyper or hypo-reactivity to sensory input or unusual interest in sensory aspects of the environment*** | **Present = 2, some/partially indicated= 1, not present= 0 for each group of behaviours listed below** |
| Parent report | Auditory | Seeking (e.g., loud music)  Avoidant (e.g., covering ears, avoidance, fear) |
|  | Tactile | Seeking (e.g., inappropriate touching, excessive tactile behaviours)  Avoidant (e.g., refusal to touch certain textures, selection of clothing based on feel) |
|  | Olfactory | Seeking (e.g., sniffing people/objects)  Avoidant (e.g., commenting/gagging) |
|  | Oral | Seeking (e.g., mouthing/chewing/licking objects/people)  Avoidant (of certain foods, flavours) |
|  | Visual | Seeking (e.g., looking at objects from unusual angles, fascination with particular visual experiences)  Avoidant (e.g., sensitivity to sunlight) |
| Diagnostic observation | Sensory behaviour | Overall level of ASD-consistent atypicality regarding sensory behaviours: pervasiveness, variety, frequency, and functional disruption of behaviours |
| **Teacher report** | |  |
| Other | Academic performance | 0 = as expected  1 = behind in a specific class  2 = behind in all classes |
| Criterion A1 | Social interest/approach | How interested is the child in his/her peers?  0 = no ASD-consistent atypicality  1 = some ASD-consistent atypicality: inconsistent interest or slight disinterest in peers  2 = significant ASD-consistent atypicality: lack of interest in peers, excessive interest/obsessions with peers |
|  | Conversation skills | Reciprocal conversation, interrupting, monologuing, conversation content  0 = no ASD-consistent atypicality  1 = some ASD-consistent atypicality: some difficulty or inconsistencies in some or all of the above  2 = significant ASD-consistent atypicality: difficulties are pervasive and interfering |
| Criterion A2 | Non-verbal interpretation | Interpretation of body language, emotions  0 = no ASD-consistent atypicality  1 = some ASD-consistent atypicality: some difficulty or inconsistencies in some or all of the above  2 = significant ASD-consistent atypicality: difficulties are pervasive and interfering |
|  | Use of nonverbal communication | Eye contact, facial expressions, gestures  0 = no ASD-consistent atypicality  1 = some ASD-consistent atypicality: some difficulty or inconsistencies in some or all of the above  2 = significant ASD-consistent atypicality: difficulties are pervasive and interfering |
| Criterion A3 | Friendship formation | To what extent is the child able to maintain friendships, negotiate conflicts, play appropriately?  0 = no ASD-consistent atypicality  1 = some ASD-consistent atypicality: inconsistencies in the above or mild difficulties  2 = significant ASD-consistent atypicality: these difficulties are pervasive and interfering |
|  | Friendship maintenance | To what extent is the child able to negotiate conflicts and maintain friendships?  0 = no ASD-consistent atypicality  1 = some ASD-consistent atypicality: inconsistencies in the above or mild difficulties  2 = significant ASD-consistent atypicality: these difficulties are pervasive and interfering |
| Criterion B1 | Stereotypical movement | 0 = no ASD-consistent atypicality  1 = some ASD-consistent atypicality, some behaviours noted but these are not interfering  2 = significant ASD-consistent atypicality, interfering behaviours noted |
|  | Stereotypical speech | 0 = no ASD-consistent atypicality  1 = some ASD-consistent atypicality, some behaviours noted but these are not interfering  2 = significant ASD-consistent atypicality, interfering behaviours noted |
|  | Stereotypical object use | 0 = no ASD-consistent atypicality  1 = some ASD-consistent atypicality, some behaviours noted but these are not interfering  2 = significant ASD-consistent atypicality, interfering behaviours noted |
| Criterion B2 | Routines and rituals | Including motor and verbal  0 = no ASD-consistent atypicality  1 = some ASD-consistent atypicality: dislikes changes in routine and rituals present  2 = significant ASD-consistent atypicality: cannot cope with changes in routine, rituals are marked and intrusive |
|  | Difficulties with change | Including difficulties with transition  0 = no ASD-consistent atypicality  1 = some ASD-consistent atypicality, sometimes has difficulty, difficulty is mild and not overly interfering  2 = significant ASD-consistent atypicality, interfering |
| Criterion B3 | Restricted interests | Has a restricted interest/preoccupation been reported?  2 = yes  1 = somewhat (clear interest reported but unclear if restricted)  0 = no |
| Criterion B4 | Sensory behaviours | 0 = no ASD-consistent atypicality  1 = some ASD-consistent atypicality, sometimes has difficulty, difficulty is mild and not overly interfering  2 = significant ASD-consistent atypicality, interfering |

**Supplementary Figure 1**

*Proportion of ASD Behaviours for Which ASD-Consistent Atypicality Was Reported by Parents*

**Supplementary Figure 2**

*Proportion of ASD Behaviours for Which ASD-Consistent Atypicality Was Observed by Diagnosticians*

**Supplementary Figure 3**

*Proportion of ASD Behaviours for Which ASD-Consistent Atypicality Was Reported by Teachers*

**Supplementary Table 2**

*Logistic Regression Predicting Behaviour by Assessment Result, Sex/Gender, and Their Interaction: Criterion A1 (all behaviours)*

|  | Effect of Ax. Result | | Effect of Sex/Gender | | Ax. Result $\times$ Sex/Gender Interaction | | Prop. Diff.  M  F  (Y-N) | ASD: M-F | | Non-ASD: M-F | |
| --- | --- | --- | --- | --- | --- | --- | --- | --- | --- | --- | --- |
| Behavioural Category | LOR [HDI_80%_] | P  ^(meaning.)^ | LOR [HDI_80%_] | P  ^(meaning.)^ | LOR [HDI_80%_] | P  ^(meaning.)^ |  | LOR  [HDI_80%_] | P  ^(meaning.)^ | LOR  [HDI_80%_] | P  ^(meaning.)^ |
| Parent report | |  |  |  |  |  |  |  |  |  |  |
| Social approach | **1.21**  **[0.78, 1.63]** | **1.00** | -0.23  [-0.66, 0.20] | -.65 | 0.86  [-0.01, 1.71] | .87 | .37  .18 | 0.20  [-0.29, 0.71] | .60 | -0.66  [-1.35, 0.05] | -.85 |
| Social norms | **1.17**  **[0.74, 1.59]** | **1.00** | -0.33  [-0.76, 0.11] | -.75 | 0.62  [-0.22, 1.49] | .78 | .34  .21 | -0.01  [-0.46, 0.43] | -.40 | -0.64  [-1.37, 0.10] | -.82 |
| Reciprocal conversation | **1.53**  **[1.08, 1.99]** | **1.00** | -0.11  [-0.57, 0.34] | -.51 | -0.61  [-1.52, 0.30] | -.77 | .29  .42 | -0.42  [-0.88, 0.02] | -.82 | 0.20  [-0.63, 0.97] | .56 |
| Sharing interests | **1.24**  **[0.64, 1.82]** | **1.00** | -0.66  [-1.26, -0.07] | -.89 | 1.23  [0.06, 2.40] | .90 | .23  .11 | -0.05  [-0.52, 0.43] | -.44 | **-1.28**  **[-2.34, -0.19]** | **-.92** |
| Sharing emotions | **0.62**  **[0.10, 1.14]** | **.91** | -0.43  [-0.96, 0.09] | -.79 | 1.11  [0.09, 2.16] | .90 | .17  .01 | 0.13  [-0.38, 0.61] | .53 | -0.99  [-1.87, -0.04] | -.89 |
| Content of conversation | **1.67**  **[1.22, 2.11]** | **1.00** | -0.00  [-0.44, 0.45] | -.39 | 0.34  [-0.53, 1.22] | .64 | .43  .36 | 0.17  [-0.30, 0.65] | .57 | -0.17  [-0.90, 0.58] | -.55 |
| Literal language | -0.13  [-0.60, 0.34] | -.53 | **-0.70**  **[-1.17, -0.23]** | **-.95** | 0.18  [-0.74, 1.12] | .54 | -.01  -.05 | -0.60  [-1.11, -0.08] | -.90 | -0.78  [-1.56, -0.00] | -.87 |
| Diagnostic Observations | |  |  |  |  |  |  |  |  |  |  |
| Social approach | **1.60**  **[1.02, 2.16]** | **1.00** | -0.15  [-0.73, 0.41] | -.54 | 0.35  [-0.76, 1.52] | .61 | .29  .25 | 0.03  [-0.41, 0.49] | .42 | -0.33  [-1.38, 0.72] | -.61 |
| Social norms | **1.13**  **[0.59, 1.65]** | **1.00** | 0.42  [-0.12, 0.95] | .79 | -0.23  [-1.29, 0.84] | -.56 | .21  .21 | 0.30  [-0.16, 0.75] | .72 | 0.53  [-0.46, 1.45] | .72 |
| Reciprocal conversation | **1.82**  **[1.36, 2.28]** | **1.00** | 0.53  [0.06, 1.00] | .89 | 0.42  [-0.50, 1.34] | .67 | .46  .37 | **0.74**  **[0.27, 1.22]** | **.96** | 0.32  [-0.49, 1.10] | .64 |
| Sharing interests | **2.43**  **[0.83, 3.97]** | **1.00** | **1.91**  **[0.35, 3.45]** | **.97** | -2.97  [-5.92, 0.19] | -.93 | .09  .11 | 0.43  [-0.19, 1.04] | .76 | 0.32  [-0.49, 1.10] | .64 |
| Sharing emotions | **1.05**  **[0.15, 1.91]** | **.94** | 0.18  [-0.71, 1.05] | .55 | -0.65  [-2.40, 1.11] | -.66 | .05  .08 | -0.14  [-0.82, 0.49] | -.53 | 0.49  [-1.13, 2.11] | .63 |
| Content of conversation | **2.31**  **[1.57, 3.02]** | **1.00** | **1.02**  **[0.28, 1.74]** | **.97** | **-2.24**  **[-3.69, -0.77]** | **-.99** | **.28**  **.53** | -0.10  [-0.55, 0.34] | -.51 | **2.16**  **[0.75, 3.54]** | **.99** |
| Literal language | **-0.93**  **[-1.57, -0.29]** | **-.95** | 0.69  [0.01, 1.36] | .88 | 0.31  [-0.97, 1.60] | .58 | -.09  -.07 | 0.86  [0.03, 1.74] | .88 | 0.53  [-0.48, 1.51] | .72 |
| Teacher report | |  |  |  |  |  |  |  |  |  |  |
| Academic achievement | -0.04  [-0.61, 0.56] | -.44 | -0.62  [-1.21, -0.03] | -.87 | 0.11  [-1.04, 1.27] | .51 | .00  -.02 | -0.56  [-1.20, 0.05] | -.83 | -0.68  [-1.67, 0.32] | -.77 |
| Social approach | 0.33  [-0.20, 0.83] | .71 | 0.50  [-0.03, 1.01] | .84 | 0.66  [-0.36, 1.69] | .76 | .16  .00 | **0.82**  **[0.28, 1.37]** | **.96** | 0.16  [-0.70, 1.03] | .53 |
| Reciprocal conversation | **1.05**  **[0.49, 1.62]** | **.99** | **0.87**  **[0.29, 1.43]** | **.97** | 0.63  [-0.50, 1.76] | .72 | .33  .15 | **1.18**  **[0.61, 1.74]** | **.99** | 0.55  [-0.45, 1.51] | .73 |

**Supplementary Table 2**

*Logistic Regression Predicting Behaviour by Assessment Result, Sex/Gender, and Their Interaction: Criterion A2 (all behaviours)*

|  | Effect of Ax. Result | | Effect of Sex/Gender | | Ax. Result $\times$ Sex/Gender Interaction | | Prop. Diff.  M  F  (Y-N) | ASD: M-F | | Non-ASD: M-F | |
| --- | --- | --- | --- | --- | --- | --- | --- | --- | --- | --- | --- |
| Behavioural Category | LOR [HDI_80%_] | P  ^(meaning.)^ | LOR [HDI_80%_] | P  ^(meaningful)^ | LOR [HDI_80%_] | P  ^(meaning.)^ |  | LOR  [HDI_80%_] | P  ^(meaning.)^ | LOR  [HDI_80%_] | P  ^(meaning.)^ |
| Parent report |  |  |  |  |  |  |  |  |  |  |  |
| Integration of verbal/NV behaviour | **3.80**  **[1.93, 5.49]** | **1.00** | **-2.08**  **[-3.73, -0.42]** | **-.96** | 2.27  [-1.03, 5.50] | .83 | .14  .26 | **-0.93**  **[-1.49, -0.40]** | **-.98** | **-3.32**  **[-6.68, -0.14]** | **-.93** |
| Eye contact | **1.06**  **[0.64, 1.49]** | **1.00** | -0.49  [-0.92, -0.06] | -.88 | 0.30  [-0.55, 1.16] | .62 | .28  .22 | -0.34  [-0.77, 0.11] | -.76 | -0.64  [-1.40, 0.08] | -.83 |
| Use of nonverbal comm. | **0.86**  **[0.32, 1.39]** | **.97** | -0.24  [-0.80, 0.29] | -.63 | 0.93  [-0.14, 2.01] | .84 | .19  .07 | 0.22  [-0.26, 0.71] | .63 | -0.71  [-1.68, 0.23] | -.79 |
| Facial expression | **1.54**  **[1.04, 2.04]** | **1.00** | **-1.17**  **[-1.67, -0.67]** | **-1.00** | 0.19  [-0.79, 1.19] | .54 | .27  .34 | **-1.07**  **[-1.52, -0.62]** | **-1.00** | **-1.26**  **[-2.15, -0.37]** | **-.95** |
| Nonverbal understand. | **1.30**  **[0.87, 1.73]** | **1.00** | -0.53  [-0.95, -0.09] | -.90 | 0.39  [-0.48, 1.24] | .67 | .35  .26 | -0.34  [-0.79, 0.13] | -.74 | -0.72  [-1.44, 0.02] | -.86 |
| Response to NV bhvr. | **1.41**  **[0.89, 1.91]** | **1.00** | -0.22  [-0.72, 0.29] | -.62 | 0.16  [-0.86, 1.15] | .53 | .28  .28 | -0.14  [-0.58, 0.30] | -.55 | -0.30  [-1.22, 0.59] | -.61 |
| Emotional regulation | **0.80**  **[0.37, 1.21]** | **.99** | 0.16  [-0.27, 0.58] | .57 | -0.27  [-1.10, 0.57] | -.60 | .16  .23 | 0.02  [-0.42, 0.46] | .41 | 0.30  [-0.43, 1.01] | -.63 |
| Diagnostic observations | |  |  |  |  |  |  |  |  |  |  |
| Eye contact | **1.35**  **[0.84, 1.83]** | **1.00** | 0.01  [-0.49, 0.51] | .41 | 0.30  [-0.67, 1.32] | .61 | .30  .24 | 0.16  [-0.28, 0.59] | .57 | -0.14  [-1.04, 0.75] | -.52 |
| Use of nonverbal comm. | **1.13**  **[0.52, 1.71]** | **.99** | -0.13  [-0.73, 0.47] | -.53 | 0.86  [-0.31, 2.06] | .80 | .21  .10 | 0.30  [-0.20, 0.78] | .70 | -0.56  [-1.65, 0.51] | -.71 |
| Facial expression | **1.14**  **[0.65, 1.61]** | **1.00** | -0.57  [-1.06, -0.08] | -.89 | 0.07  [-0.86, 1.06] | .49 | .21  .25 | -0.53  [-0.97, -0.08] | -.89 | -0.61  [-1.47, 0.26] | -.77 |
| Nonverbal understand. | **1.12**  **[0.64, 1.57]** | **1.00** | 0.12  [-0.35, 0.59] | .52 | 0.57  [-0.35, 1.51] | .74 | .31  .18 | 0.40  [-0.04, 0.84] | .81 | -0.17  [-0.99, 0.65] | -.54 |
| Teacher report | |  |  |  |  |  |  |  |  |  |  |
| Use of nonverbal comm. | 0.65  [0.01, 1.25] | .89 | **0.99**  **[0.34, 1.60]** | **.96** | -0.53  [-1.77, 0.72] | -.68 | .09  .15 | **0.72**  **[0.13, 1.27]** | **.92** | **1.25**  **[0.13, 2.35]** | **.92** |
| Nonverbal understand. | **1.30**  **[0.70, 1.88]** | **1.00** | 0.61  [0.02, 1.20] | .87 | 1.07  [-0.09, 2.28] | .85 | .43  .17 | **1.14**  **[0.57, 1.73]** | **.99** | 0.07  [-0.94, 1.10] | .49 |

**Supplementary Table 3**

*Logistic Regression Predicting Behaviour by Assessment Result, Sex/Gender, and Their Interaction: Criterion A3 (all behaviours)*

|  | Effect of Ax. Result | Effect of Sex/Gender | Ax. Result $\times$ Sex/Gender Interaction | Prop. Diff.  M  F  (Y-N) | ASD: M-F | Non-ASD: M-F |
| --- | --- | --- | --- | --- | --- | --- |

Criterion A3

|  | Effect of Ax. Result | | Effect of Sex/Gender | | Ax. Result $\times$ Sex/Gender Interaction | | Prop. Diff.  M  F  (Y-N) | ASD: M-F | | Non-ASD: M-F | |
| --- | --- | --- | --- | --- | --- | --- | --- | --- | --- | --- | --- |
| Behavioural Category | LOR [HDI_80%_] | P  ^(meaning.)^ | LOR [HDI_80%_] | P  ^(meaningful)^ | LOR [HDI_80%_] | P  ^(meaning.)^ |  | LOR  [HDI_80%_] | P  ^(meaning.)^ | LOR  [HDI_80%_] | P  ^(meaning.)^ |
| Parent report |  |  |  |  |  |  |  |  |  |  |  |
| Adjusting behaviour for situation | **1.38**  **[0.73, 2.00]** | **1.00** | 0.07  [-0.57, 0.69] | .48 | -0.21  [-1.46, 1.05] | -.55 | .19  .21 | -0.04  [-0.52, 0.44] | -.43 | 0.18  [-0.99, 1.35] | .53 |
| Imaginative play | **0.88**  **[0.35, 1.38]** | **.98** | 0.54  [0.02, 1.06] | .87 | -0.43  [-1.44, 0.64] | -.66 | .14  .18 | 0.33  [-0.11, 0.80] | .74 | 0.75  [-0.23, 1.66] | .83 |
| Submissive/  dominating in play | 0.28  [-0.13, 0.71] | .71 | **-0.69**  **[-1.11, -0.25]** | **-.96** | 0.14  [-0.70, 0.99] | .53 | .08  .05 | **-0.61**  **[-1.07, -0.18]** | **-.93** | -0.76  [-1.46, -0.01] | -.88 |
| Possessive/ losing | **0.80**  **[0.38, 1.23]** | **.98** | 0.19  [-0.24, 0.62] | .61 | 0.79  [-0.07, 1.65] | .85 | .28  .09 | **0.58**  **[0.15, 1.03]** | **.92** | -0.22  [-0.94, 0.53] | -.57 |
| Friendship formation | **1.44**  **[1.01, 1.88]** | **1.00** | -0.07  [-0.51, 0.37] | -.47 | **1.39**  **[0.53, 2.27]** | **.97** | **.48**  **.18** | **0.62**  **[0.16, 1.08]** | **.93** | -0.76  [-1.49, -0.00] | -.87 |
| Friendship maintenance | **1.41**  **[0.98, 1.83]** | **1.00** | -0.17  [-0.59, 0.27] | -.58 | 0.51  [-0.34, 1.40] | .73 | .39  .27 | 0.09  [-0.39, 0.57] | .49 | -0.43  [-1.14, 0.27] | -.72 |
| Social motivation | 0.15  [-0.37, 0.67] | .55 | 0.12  [-0.40, 0.64] | .52 | 0.19  [-0.83, 1.25] | .55 | .04  .01 | 0.22  [-0.31, 0.75] | .61 | 0.02  [-0.90, 0.89] | .46 |
| Consistent companions | **0.68**  **[0.21, 1.13]** | **.95** | 0.18  [-0.29, 0.64] | .59 | -0.04  [-0.94, 0.90] | -.47 | .18  .14 | 0.16 [-0.29, 0.61] | .57 | 0.19 [-0.60, 1.01] | .56 |
| Diagnostic observations | |  |  |  |  |  |  |  |  |  |  |
| Friendship understand. | **1.01**  **[0.58, 1.45]** | **1.00** | 0.05  [-0.41, 0.49] | .44 | -0.74  [-1.63, 0.16,] | -.82 | .15  .31 | -0.32  [-0.75, 0.12] | -.74 | 0.41  [-0.37, 1.21] | .70 |
| Inclusiveness in play | **1.79**  **[0.95, 2.60]** | **1.00** | 0.38  [-0.47, 1.17] | .67 | **-1.85**  **[-3.46, -0.18]** | **-.93** | **.08**  **.22** | -0.55  [-1.11, -0.02] | -.85 | 1.29  [-0.23, 2.84] | .86 |
| Imaginative/ spont. play | **1.38**  **[0.56, 2.17]** | **.99** | 0.06  [-0.74, 0.86] | .47 | **1.88**  **[0.29, 3.47]** | **.92** | **.39**  **.07** | **0.99**  **[0.39, 1.60]** | **.97** | -0.89  [-2.32, 0.63] | -.76 |
| Teacher report | |  |  |  |  |  |  |  |  |  |  |
| Friendship formation | **0.99**  **[0.44, 1.51]** | **.99** | 0.55  [0.01, 1.09] | .86 | 0.43  [-0.63, 1.52] | .65 | .29  .17 | **0.76**  **[0.21, 1.33]** | **.94** | 0.34  [-0.59, 1.24] | .63 |
| Friendship maintenance | **0.96**  **[0.44, 1.48]** | **.98** | 0.25  [-0.28, 0.77] | .64 | 0.60  [-0.44, 1.66] | .73 | .30  .16 | 0.55  [-0.02, 1.10] | .85 | -0.05  [-0.95, 0.81] | -.47 |

**Supplementary Table 4**

*Logistic Regression Predicting Behaviour by Assessment Result, Sex/Gender, and Their Interaction: Criterion B1 (all behaviours – fine grained)*

|  | Effect of Ax. Result | | Effect of Sex/Gender | | Ax. Result $\times$ Sex/Gender Interaction | | Prop. Diff.  M  F  (Y-N) | ASD: M-F | | Non-ASD: M-F | |
| --- | --- | --- | --- | --- | --- | --- | --- | --- | --- | --- | --- |
| Behavioural Category | LOR [HDI_80%_] | P  ^(meaning.)^ | LOR [HDI_80%_] | P  ^(meaningful)^ | LOR [HDI_80%_] | P  ^(meaning.)^ |  | LOR  [HDI_80%_] | P  ^(meaning.)^ | LOR  [HDI_80%_] | P  ^(meaning.)^ |
| Motor stereotypies | 1.10  [0.58, 1.61] | **1.00** | 0.34  [-0.19, 0.84] | .73 | -0.82  [-1.82, 0.24] | -.82 | .15  .28 | -0.07  [-0.51, 0.38] | -.47 | 0.73  [-0.20, 1.65] | .82 |
| Toe walking | 0.79  [-0.01, 1.54] | .90 | 0.76  [-0.03, 1.53] | .89 | **-1.96**  **[-3.48, -0.38]** | **-.96** | **-.03**  **.15** | -0.22  [-0.77, 0.36] | -.61 | **1.74**  **[0.27, 3.15]** | **.96** |
| Flapping | 0.76  [0.09, 1.38] | .92 | -0.07  [-0.75, 0.56] | -.48 | -0.51  [-1.66, 0.81] | -.66 | .06  .12 | -0.33  [-0.89, 0.23] | -.70 | 0.17  [-1.05, 1.30] | .53 |
| Spinning | 0.73  [-0.13, 1.57] | .85 | 0.07  [-0.80, 0.92] | .48 | -1.59  [-0.80, 0.18] | -.88 | -.01  .09 | -0.72  [-1.39, 0.00] | -.88 | 0.85  [-0.75, 2.39] | .74 |
| Gross motor mannerism | -0.14  [-0.71, 0.39] | -.54 | -0.34  [-0.91, 0.22] | -.71 | -0.15  [-1.27, 0.94] | -.52 | -.02  -.01 | -0.41  [-1.02, 0.22] | -.74 | -0.27  [-1.21, 0.64] | -.59 |
| Rocking/ jumping | **0.69**  **[0.13, 1.23]** | **.92** | -0.12  [-0.69, 0.43] | -.52 | 1.03  [-0.03, 2.15] | .87 | .09  .02 | 0.39  [-0.13, 0.91] | .77 | -0.65  [-1.61, 0.35] | -.76 |
| Rigidity | 0.65  [-0.06, 1.31] | .86 | -0.35  [-1.04, 0.34] | -.68 | -0.51  [-1.88, 0.85] | -.66 | .03  .09 | -0.59  [-1.22, 0.00] | -.85 | -0.09  [-1.32, 1.12] | -.50 |
| Hand mannerisms | 0.04  [-0.46, 0.59] | .44 | 0.11  [-0.42, 0.64] | .51 | 0.50  [-0.58, 1.53] | .69 | .04  -.03 | 0.35  [-0.20, 0.92] | .72 | -0.14  [-1.03, 0.75] | -.52 |
| Self-injurious | **0.72**  **[0.15, 1.28]** | **.93** | 0.04  [-0.53, 0.62] | .45 | 0.27  [-0.84, 1.43] | .57 | .12  .08 | 0.18  [-0.35, 0.68] | .58 | -0.09  [-1.11, 0.91] | -.49 |
| Repetitive body use | **-0.84**  **[-1.46, -0.21]** | **-.93** | -0.65  [-1.31, -0.03] | -.87 | -0.37  [-1.64, 0.85] | -.61 | -.07  -.08 | -0.84  [-1.66, 0.03] | -.88 | -0.48  [-1.41, 0.46] | -.70 |
| Speech/ language | **3.38**  **[1.95, 4.72]** | **1.00** | **2.28**  **[0.87, 3.63]** | **1.00** | **-3.63**  **[-6.29, -0.80]** | **-.99** | **.34**  **.44** | 0.47  [0.02, 0.91] | .86 | **4.05**  **[1.26, 6.74]** | **1.00** |
| Echolalia | **0.97**  **[0.11, 1.75]** | **.94** | **0.95**  **[0.12, 1.75]** | **.93** | -0.81  [-2.42, 0.81] | -.72 | .07  .08 | 0.54  [-0.06, 1.13] | .83 | 1.33  [-0.19, 2.84] | .88 |
| Third person referencing | 0.15  [-0.87, 1.15] | .53 | -0.67  [-1.66, 0.34] | -.78 | **1.77**  **[0.26, 3.67]** | **.87** | **.02**  **-.04** | 0.19  [-0.86, 1.20] | .54 | -1.55  [-3.26, 0.11] | -.88 |
| Neologisms | 0.43  [-0.10, 0.97] | .80 | **-0.85**  **[-1.39, -0.31]** | **-.97** | **1.37**  **[0.28, 2.42]** | **.94** | **.13**  **-.05** | -0.17  [-0.68, 0.35] | -.57 | **-1.54**  **[-2.46, -0.59]** | **-.98** |
| Pronoun reversal | 0.40  [-0.45, 1.17] | .67 | 0.58  [-0.27, 1.35] | .79 | -1.76  [-3.30, -0.07] | -.93 | -.05  .08 | -0.29  [-0.98, 0.39] | -.64 | 1.42  [-0.05, 2.86] | .91 |
| Repetitive speech | **0.99**  **[0.47, 1.49]** | **.99** | **0.99**  **[0.47, 1.49]** | **.99** | -0.97  [-1.99, 0.05] | -.87 | .12  .27 | 0.50  [0.05, 0.93] | .88 | **1.47**  **[0.55, 2.38]** | **.98** |
| Accents | **1.14**  **[0.43, 1.84]** | **.98** | **-1.25**  **[-1.94, -0.53]** | **-.98** | 0.29  [-1.11, 1.69] | .57 | .09  .15 | **-1.09**  **[-1.67, -0.54]** | **-.99** | **-1.39**  **[-2.67, -0.12]** | **-.91** |
| Unusual noises | **0.98**  **[0.49, 1.47]** | **.99** | 0.38  [-0.11, 0.89] | .77 | 0.73  [-0.23, 1.73] | .79 | .28  .11 | **0.74**  **[0.30, 1.21]** | **.97** | 0.02  [-0.89, 0.87] | .45 |
| Talking to self | -0.17  [-1.29, 0.87] | -.53 | **-1.35**  **[-2.42, -0.23]** | **-.94** | 0.68  [-1.40, 2.82] | .64 | .00  -.03 | -0.98  [-2.23, 0.20] | -.84 | -1.69  [-3.42, 0.04] | -.90 |
| Odd prosody | **1.24**  **[0.51, 1.97]** | **.99** | **1.12**  **[0.36, 1.82]** | **.98** | **-2.75**  **[-4.15, -1.26]** | **-1.00** | **-.03**  **.33** | -0.25  [-0.73, 0.20] | -.66 | **2.50**  **[1.10, 3.87]** | **1.00** |
| Object use | 1.36  [0.72, 1.99] | **1.00** | 0.07  [-0.57, 0.69] | .48 | -0.71  [-1.95, 0.56] | -.74 | .18  .24 | -0.28  [-0.76, 0.20] | -.69 | 0.42  [-0.72, 1.59] | .64 |
| Lining up | -0.14  [-0.56, 0.28] | -.55 | 0.31  [-1.19, 0.73] | .73 | 0.33  [-0.50, 1.19] | .64 | .01  -.07 | 0.47  [0.02, 0.92] | .85 | 0.14  [-0.56, 0.88] | .53 |
| Grouping | 0.07  [-0.43, 0.56] | .47 | -0.45  [-0.94, 0.06] | -.82 | 0.01  [-0.99, 1.00] | .45 | .00  .01 | -0.46  [-0.98, 0.08] | -.81 | -0.45  [-1.32, 0.38] | -.70 |
| Spinning/ flicking/ pushing | **1.92**  **[0.96, 2.80]** | **1.00** | 0.30  [-0.59, 1.20] | .62 | 0.55  [-1.27, 2.31] | .63 | .21  .12 | 0.57  [0.04, 1.13] | .87 | 0.02  [-1.76, 1.67] | .48 |
| Repetitive play | 0.55  [-0.15, 1.22] | .83 | -0.61  [-1.28, 0.08] | -.83 | 0.43  [-0.91, 1.80] | .62 | .04  .03 | -0.39  [-1.02, 0.23] | -.73 | -0.83  [-2.03, 0.36] | -.79 |
| Deconstruct-ion | **3.22**  **[1.45, 4.86]** | **1.00** | -0.78  [-2.38, 0.81] | -.72 | **4.11**  **[0.88, 7.20]** | **.97** | **.24**  **.05** | -0.39  [-1.02, 0.23] | -.73 | -0.83  [-2.03, 0.36] | -.79 |

**Supplementary Table 5**

*Logistic Regression Predicting Behaviour by Assessment Result, Sex/Gender, and Their Interaction: Criterion B1 (all behaviours – domain based)*

|  | Effect of Ax. Result | | Effect of Sex/Gender | | Ax. Result $\times$ Sex/Gender Interaction | | Prop. Diff.  M  F  (Y-N) | ASD: M-F | | Non-ASD: M-F | |
| --- | --- | --- | --- | --- | --- | --- | --- | --- | --- | --- | --- |
| Behavioural Category | LOR [HDI_80%_] | P  ^(meaning.)^ | LOR [HDI_80%_] | P  ^(meaningful)^ | LOR [HDI_80%_] | P  ^(meaning.)^ |  | LOR  [HDI_80%_] | P  ^(meaning.)^ | LOR  [HDI_80%_] | P  ^(meaning.)^ |
| Diagnostic observations | |  |  |  |  |  |  |  |  |  |  |
| Stereotypical movement | 0.73  [-0.17, 1.56] | .84 | 0.53  [-0.33, 1.40] | .75 | -0.61  [-2.34, 1.09] | -.66 | .14  .28 | 0.23  [-0.47, 0.92] | .59 | 0.80  [-0.80, 2.31] | .74 |
| Stereotypical speech/lang. | **1.06**  **[0.38, 1.72]** | **.98** | -0.06  [-0.73, 0.61] | -.47 | 0.00  [-1.33, 1.34] | -.46 | .34  .44 | -0.05  [-0.59, 0.48] | -.46 | -0.08  [-1.32, 1.13] | -.49 |
| Teacher report | |  |  |  |  |  |  |  |  |  |  |
| Stereotypical movement | 0.43  [-0.31, 1.15] | .72 | 0.61  [-0.14, 1.34] | .82 | 1.05  [-0.42, 2.51] | .79 | .14  .00 | **1.12**  **[0.33, 1.88]** | **.96** | 0.08  [-1.16, 1.33] | .49 |
| Stereotypical speech/lang. | **1.02**  **[0.17, 1.81]** | **.95** | **1.20**  **[0.37, 2.03]** | **.97** | -0.35  [-1.97, 1.31] | -.58 | .18  .12 | **1.01**  **[0.35, 1.68]** | **.96** | **1.36**  **[-0.19, 2.80]** | **.89** |
| Stereotypical object use | -0.92  [-2.65, 0.79] | -.74 | **2.49**  **[0.64, 4.31]** | **.98** | **4.34**  **[1.09, 7.72]** | **.97** | .10  -.04 | **4.54**  **[1.66, 7.35]** | **1.00** | 0.29  [-1.62, 2.13] | .55 |

**Supplementary Table 6**

*Logistic Regression Predicting Behaviour by Assessment Result, Sex/Gender, and Their Interaction: Criterion B2 (all behaviours)*

|  | Effect of Ax. Result | | Effect of Sex/Gender | | Ax. Result $\times$ Sex/Gender Interaction | | Prop. Diff.  M  F  (Y-N) | ASD: M-F | | Non-ASD: M-F | |
| --- | --- | --- | --- | --- | --- | --- | --- | --- | --- | --- | --- |
| Behavioural Category | LOR [HDI_80%_] | P  ^(meaning.)^ | LOR [HDI_80%_] | P  ^(meaningful)^ | LOR [HDI_80%_] | P  ^(meaning.)^ |  | LOR  [HDI_80%_] | P  ^(meaning.)^ | LOR  [HDI_80%_] | P  ^(meaning.)^ |
| Parent report |  |  |  |  |  |  |  |  |  |  |  |
| Distress at change | **1.33**  **[0.89, 1.75]** | **1.00** | -0.37  [-0.81, 0.07] | -.79 | **1.34**  **[0.48, 2.21]** | **.97** | **.46**  **.16** | 0.30  [-0.17, 0.76] | .70 | **-1.04**  **[-1.78, -0.32]** | **-.95** |
| Routine adherence | **1.04**  **[0.60, 1.47]** | **1.00** | **-0.61**  **[-1.05, -0.16]** | **-.93** | 0.83  [-0.04, 1.71] | .86 | .31  .15 | -0.19  [-0.62, 0.25] | -.61 | **-1.03**  **[-1.79, -0.26]** | **-.94** |
| Task switching/ transitioning | **1.41**  **[0.90, 1.90]** | **1.00** | -0.27  [-0.78, 0.23] | -.66 | 0.09  [-0.90, 1.11] | .50 | .27  .28 | -0.22  [-0.66, 0.21] | -.63 | -0.32  [-1.26, 0.56] | -.62 |
| Cognitive rigidity | **1.82**  **[1.36, 2.27]** | **1.00** | -0.21  [-0.66, 0.26] | -.62 | **1.12**  **[0.23, 2.04]** | **.93** | **.53**  **.30** | 0.35  [-0.13, 0.80] | .75 | -0.77  [-1.55, 0.00] | -.86 |
| Diagnostic observations | |  |  |  |  |  |  |  |  |  |  |
| Routine adherence | 0.59  [-0.52, 1.57] | .73 | 0.57  [-0.47, 1.60] | .73 | 0.21  [-1.85, 2.25] | .53 | .01  .01 | 0.65  [-0.32, 1.60] | .78 | 0.48  [-1.31, 2.31] | .61 |
| Cognitive rigidity | **1.31**  **[0.61, 2.00]** | **.99** | -0.48  [-1.18, 0.22] | -.76 | -0.11  [-1.49, 1.27] | -.51 | .11  .17 | -0.54  [-1.07, -0.01] | -.86 | -0.43  [-1.74, 0.82] | -.63 |
| Teacher report | |  |  |  |  |  |  |  |  |  |  |
| Distress at change | 0.63  [0.06, 1.20] | .89 | 0.44  [-0.14, 1.01] | .79 | 0.26  [-0.85, 1.42] | .57 | .17  .10 | 0.57  [0.01, 1.13] | .86 | 0.32  [-0.67, 1.31] | .61 |

**Supplementary Table 7**

*Logistic Regression Predicting Behaviour by Assessment Result, Sex/Gender, and Their Interaction: Criterion B3 (all behaviours – fine grained)*

|  | Effect of Ax. Result | | Effect of Sex/Gender | | Ax. Result $\times$ Sex/Gender Interaction | | Prop. Diff.  M  F  (Y-N) | ASD: M-F | | Non-ASD: M-F | |
| --- | --- | --- | --- | --- | --- | --- | --- | --- | --- | --- | --- |
| Restricted Interest | LOR [HDI_80%_] | P  ^(meaning.)^ | LOR [HDI_80%_] | P  ^(meaningful)^ | LOR [HDI_80%_] | P  ^(meaning.)^ |  | LOR  [HDI_80%_] | P  ^(meaning.)^ | LOR  [HDI_80%_] | P  ^(meaning.)^ |
| Specific program/ character | 0.18  [-0.27, 0.62] | .60 | -0.03  [-0.48, 0.43] | -.42 | **-1.25**  **[-2.17, -0.36]** | -.95 | -.01  .18 | **-0.65**  **[-1.10, -0.18]** | **-.94** | 0.59  [-0.20, 1.36] | .80 |
| Random objects | **0.72**  **[0.27, 1.17]** | **.97** | -0.44  [-0.91, -0.00] | -.84 | **1.43**  **[0.56, 2.33]** | **.97** | **.29**  **.00** | 0.27  [-0.18, 0.70] | .69 | **-1.16**  **[-1.92, -0.37]** | **-.96** |
| Vehicles | -0.90  [-1.67, -0.02] | -.90 | **2.17**  **[1.31, 2.99]** | **1.00** | **3.38**  **[1.73, 5.02]** | **1.00** | **.15**  **-.12** | **3.77**  **[2.41, 5.00]** | **1.00** | 0.46  [-0.51, 1.46] | .68 |
| Toys | 0.54  [0.06, 1.00] | .89 | 0.35  [-0.13, 0.82] | .75 | -0.90  [-1.85, 0.05] | -.87 | .02  .20 | -0.10  [-0.55, 0.35] | -.50 | 0.79  [-0.06, 1.61] | .86 |
| Screens | **1.49**  **[0.73, 2.27]** | **1.00** | **1.96**  **[1.15, 2.73]** | **1.00** | -0.61  [-2.07, 0.97] | -.67 | .27  .14 | **1.65**  **[1.11, 2.16]** | **1.00** | **2.25**  **[0.82, 3.70]** | **.99** |
| Animals | **1.48**  **[0.67, 2.25]** | **1.00** | 0.44  [-0.38, 1.18] | .72 | -1.50  [-2.99, 0.11] | -.90 | .10  .24 | -0.31  [-0.82, 0.18] | -.70 | 1.19  [-0.31, 2.64] | .85 |
| Systems | **1.78**  **[0.67, 2.83]** | **.99** | -0.25  [-1.29, 0.82] | -.57 | 0.86  [-1.24, 2.90] | .69 | .11  .08 | 0.18  [-0.48, 0.86] | .56 | -0.75  [-2.70, 1.27] | -.67 |
| Craft | 0.72  [0.07, 1.38] | .90 | **-0.94**  **[-1.59, -0.28]** | **-.95** | -0.09  [-1.40, 1.19] | -.50 | .05  .11 | **-0.97**  **[-1.58, -0.35]** | **-.97** | -0.88  [-2.06, 0.24] | -.81 |
| Sport/activity | -0.43  [-0.96, 0.11] | -.78 | -0.21  [-0.77, 0.34] | -.60 | 0.69  [-0.36, 1.78] | .76 | -.01  -.11 | 0.13  [-0.52, 0.77] | .53 | -0.56  [-1.44, 0.30] | -.75 |
| People | **2.83**  **[0.94, 4.67]** | **.99** | **-2.18**  **[-3.90, -0.42]** | **-.96** | 2.43  [-0.99, 5.76] | .84 | .05  .09 | **-0.94**  **[-1.79, -0.10]** | **-.91** | -3.26  [-6.50, 0.07] | -.92 |

|  | Effect of Ax. Result | | Effect of Sex/Gender | | Ax. Result $\times$ Sex/Gender Interaction | | Prop. Diff.  M  F  (Y-N) | ASD: M-F | | Non-ASD: M-F | |
| --- | --- | --- | --- | --- | --- | --- | --- | --- | --- | --- | --- |
| Source | LOR [HDI_80%_] | P  ^(meaning.)^ | LOR [HDI_80%_] | P  ^(meaningful)^ | LOR [HDI_80%_] | P  ^(meaning.)^ |  | LOR  [HDI_80%_] | P  ^(meaning.)^ | LOR  [HDI_80%_] | P  ^(meaning.)^ |
| Diagnostic observations | **3.14**  **[1.62, 4.57]** | **1.00** | **2.18**  **[0.66, 3.60]** | **1.00** | -3.01  [-5.86, -0.06] | -.95 | .12  .15 | **0.67**  **[0.19, 1.13]** | **.94** | **3.63**  **[0.74, 6.45]** | **.98** |
| Teacher report | **1.19**  **[0.55, 1.83]** | **.99** | **1.05**  **[0.40, 1.69]** | **.98** | 0.41  [-0.85, 1.69] | .63 | .33  .16 | **1.25**  **[0.65, 1.83]** | **.99** | 0.83  [-0.33, 1.93] | .81 |

**Supplementary Table 8**

*Logistic Regression Predicting Behaviour by Assessment Result, Sex/Gender, and Their Interaction: Criterion B4 (all behaviours, parent report)*

|  | Effect of Ax. Result | | Effect of Sex/Gender | | Ax. Result $\times$ Sex/Gender Interaction | | Prop. Diff.  M  F  (Y-N) | ASD: M-F | | Non-ASD: M-F | |
| --- | --- | --- | --- | --- | --- | --- | --- | --- | --- | --- | --- |
| Behavioural Category | LOR [HDI_80%_] | P  ^(meaning.)^ | LOR [HDI_80%_] | P  ^(meaningful)^ | LOR [HDI_80%_] | P  ^(meaning.)^ |  | LOR  [HDI_80%_] | P  ^(meaning.)^ | LOR  [HDI_80%_] | P  ^(meaning.)^ |
| Auditory: seeking | 0.44  [-0.27, 1.12] | .74 | 0.03  [-0.68, 0.72] | .45 | 0.19  [-1.21, 1.55] | .53 | .05  .03 | 0.12  [-0.54, 0.76] | .52 | -0.06  [-1.30, 1.18] | -.48 |
| Auditory: avoiding | **1.40**  **[0.97, 1.82]** | **1.00** | -0.09  [-0.54, 0.33] | -.49 | 0.86  [-0.00, 1.71] | .87 | .43  .23 | 0.33  [-0.14, 0.78] | .74 | -0.52  [-1.24, 0.22] | -.77 |
| Tactile:  seeking | **1.46**  **[0.94, 1.97]** | **1.00** | 0.38  [-0.15, 0.89] | .76 | -0.52  [-1.56, 0.50] | -.71 | .27  .33 | 0.12  [-0.32, 0.55] | .52 | 0.64  [-0.32, 1.56] | .78 |
| Tactile: avoiding | **1.20**  **[0.77, 1.62]** | **1.00** | -0.44  [-0.87, -0.01] | -.84 | -0.02  [-0.86, 0.85] | -.45 | .18  .29 | -0.45  [-0.91, 0.00] | -.84 | -0.43  [-1.16, 0.29] | -.72 |
| Olfactory: seeking | **1.29**  **[0.41, 2.14]** | **.98** | 0.17  [-0.70, 1.03] | .55 | -0.57  [-2.23, 1.17] | -.64 | .08  .11 | -0.10  [-0.72, 0.50] | -.50 | 0.44  [-1.17, 2.02] | .61 |
| Olfactory: avoiding | **1.71**  **[0.94, 2.45]** | **1.00** | -0.61  [-1.38, 0.12] | -.81 | 0.53  [-0.96, 2.01] | .65 | .19  .17 | -0.35  [-0.85, 0.16] | -.74 | -0.88  [-2.30, 0.47] | -.77 |
| Oral:  seeking | **1.09**  **[0.62, 1.55]** | **1.00** | -0.34  [-0.81, 0.13] | -.74 | -0.33  [-1.27, 0.60] | -.62 | .19  .28 | -0.50  [-0.94, -0.05] | -.88 | -0.18  [-1.00, 0.65] | -.55 |
| Oral:  avoiding | **1.04**  **[0.52, 1.54]** | **.99** | -0.33  [-0.84, 0.19] | -.71 | **1.35**  **[0.32, 2.36]** | **.94** | **.29**  **.07** | 0.34  [-0.10, 0.82] | .75 | -0.99  [-1.93, -0.10] | -.89 |
| Visual:  seeking | **0.71**  **[0.19, 1.25]** | **.94** | -0.61  [-1.14, -0.08] | -.89 | 0.82  [-0.22, 1.88] | .81 | .15  .06 | -0.20  [-0.69, 0.29] | -.73 | **-1.02**  **[-1.97, -0.10]** | **-.90** |
| Visual: avoiding | **0.96**  **[0.11, 1.77]** | **.93** | **-1.05**  **[-1.86, -0.21]** | **-.95** | **2.88**  **[1.21, 4.45]** | **.99** | **.14**  **-.06** | 0.39  [-0.23, 1.01] | .73 | **-2.47**  **[-4.00, -0.99]** | **-.99** |

*Note.* Positive LORs (ASD assessment result) = greater probability of being reported if the assessment result was positive for ASD; Positive LORs (sex/gender) = greater probability of being reported for males. Differences in boldface indicate the HDI_80%_ lay entirely outside the ROPE. P_(meaningful)_ indicates the probability that the true difference fell outside the ROPE and in the observed direction. Prop. Diff. = proportion of children with behaviour reported for males (Yes - No ASD result) and females (Yes - No ASD result). LOR = log odds ratio.
